# Supplementary material for: Regulation of cellular cholesterol distribution via non-vesicular lipid transport at ER-Golgi contact sites
Source: Nat Commun. 2023 Sep 21;14:5867. doi: 10.1038/s41467-023-41213-w (PMC10514280; doi:10.1038/s41467-023-41213-w)
Supplement: Supplementary file 1 — Supplementary Information [file 41467_2023_41213_MOESM1_ESM.pdf]

Supplementary Information

Regulation of cellular cholesterol distribution via non-vesicular lipid transport at ER-Golgi contact sites

Tomoki Naito[1], Haoning Yang[1], Dylan Hong Zheng Koh[1], Divyanshu Mahajan[2], Lei Lu[2], Yasunori Saheki[1,3]*

[1]Lee Kong Chian School of Medicine, Nanyang Technological University, 308232, Singapore

[2]School of Biological Sciences, Nanyang Technological University, Singapore, 637551, Singapore

[3]Institute of Resource Development and Analysis, Kumamoto University, Kumamoto, 860-0811, Japan

*Address correspondence to: yasunori.saheki@ntu.edu.sg (Y.S.)

- **Supplementary Figures 1-10**
- **Supplementary Table 1**

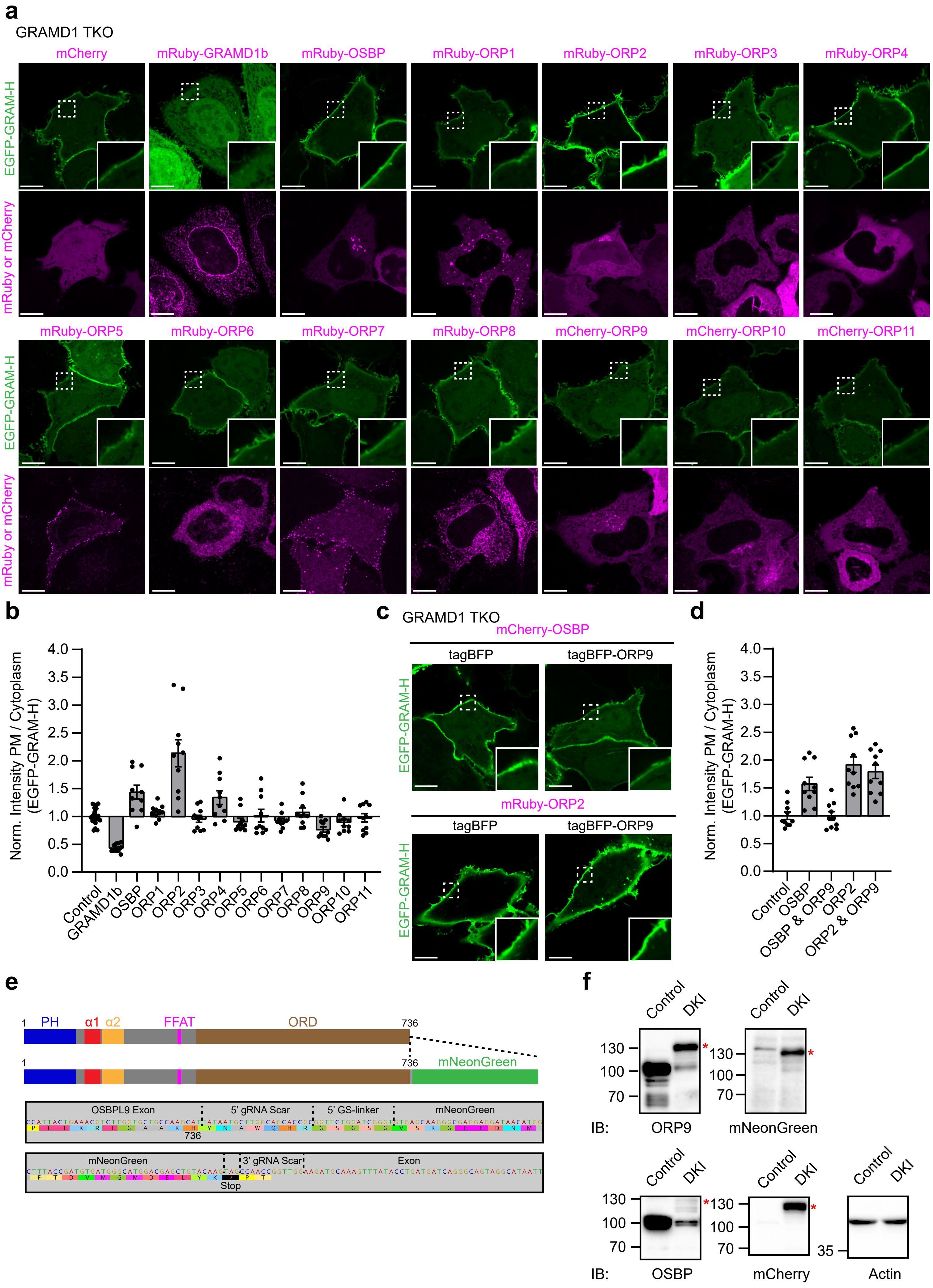

a
GRAMD1 TKO
mCherry
mRuby-GRAMD1b
mRuby-OSBP
mRuby-ORP1
mRuby-ORP2
mRuby-ORP3
mRuby-ORP4
EGFP-GRAM-H
mRuby or mCherry
mRuby-ORP5
mRuby-ORP6
mRuby-ORP7
mRuby-ORP8
mCherry-ORP9
mCherry-ORP10
mCherry-ORP11
b
Norm. Intensity PM / Cytoplasm (EGFP-GRAM-H)
Control
GRAMD1b
OSBP
ORP1
ORP2
ORP3
ORP4
ORP5
ORP6
ORP7
ORP8
ORP9
ORP10
ORP11
c
GRAMD1 TKO
mCherry-OSBP
tagBFP
tagBFP-ORP9
mRuby-ORP2
d
Control
OSBP
OSBP & ORP9
ORP2
ORP2 & ORP9
e
PH
α1
α2
FFAT
ORD
736
mNeonGreen
OSBPL9 Exon
5' gRNA Scar
5' GS-linker
3' gRNA Scar
Exon
Stop
f
Control
DKI
IB:
ORP9
mNeonGreen
OSBP
mCherry
Actin

Supplementary Figure 1. ORP9 regulates the abundance of accessible cholesterol in the PM. a. Confocal images of live GRAMD1 TKO HeLa cells expressing EGFP-GRAM-H (accessible cholesterol biosensor) together with either mCherry control, mRuby-tagged GRAMD1b (mRuby-GRAMD1b), or mRuby/mCherry-tagged ORP family proteins as indicated. Insets show at higher magnification the regions around the plasma membrane (PM) indicated by white dashed boxes. Scale bars, 10 μm. **b.** Quantification of the ratio of PM signals to the cytosolic signals of EGFP-GRAM-H as shown in (**a**) [mean ± SEM, n = 20 cells (Control); n = 10 cells (all other conditions); data are pooled from one experiment]. **c.** Confocal images of live GRAMD1 TKO HeLa cells expressing EGFP-GRAM-H together with indicated combination of proteins. Insets show at higher magnification the regions around the PM as indicated by white dashed boxes. Scale bars, 10 μm. **d.** Quantification of the ratio of PM signals to the cytosolic signals of EGFP-GRAM-H as shown in (**c**) (mean ± SEM, n = 10 cells for each condition; data are pooled from one experiment). **e.** Cartoon diagram showing the endogenously edited locus of endoORP9-mNeonGreen in HeLa cells. ORP9 (top) and edited endoORP9-mNeonGreen (bottom) are shown. DNA scar as a result of the homology-independent targeted integration, GS linker, and mNeonGreen sequence are indicated. **f.** Lysates of control wild-type (WT) HeLa cells and double knock-in (DKI) HeLa cells, in which mNeonGreen was tagged to the C-terminus of ORP9 (endoORP9-mNG) and a mScarlet-I was tagged to the N-terminus of OSBP (mSc-endoOSBP), were processed by SDS-PAGE and immunoblotting (IB) with anti-ORP9, anti-mNeonGreen, anti-OSBP, anti-mCherry (also recognizing mScarlet-I), and anti-actin antibodies. Note the presence of tagged proteins (marked by red asterisks) only in DKI cells. Source data are provided as a Source Data file.

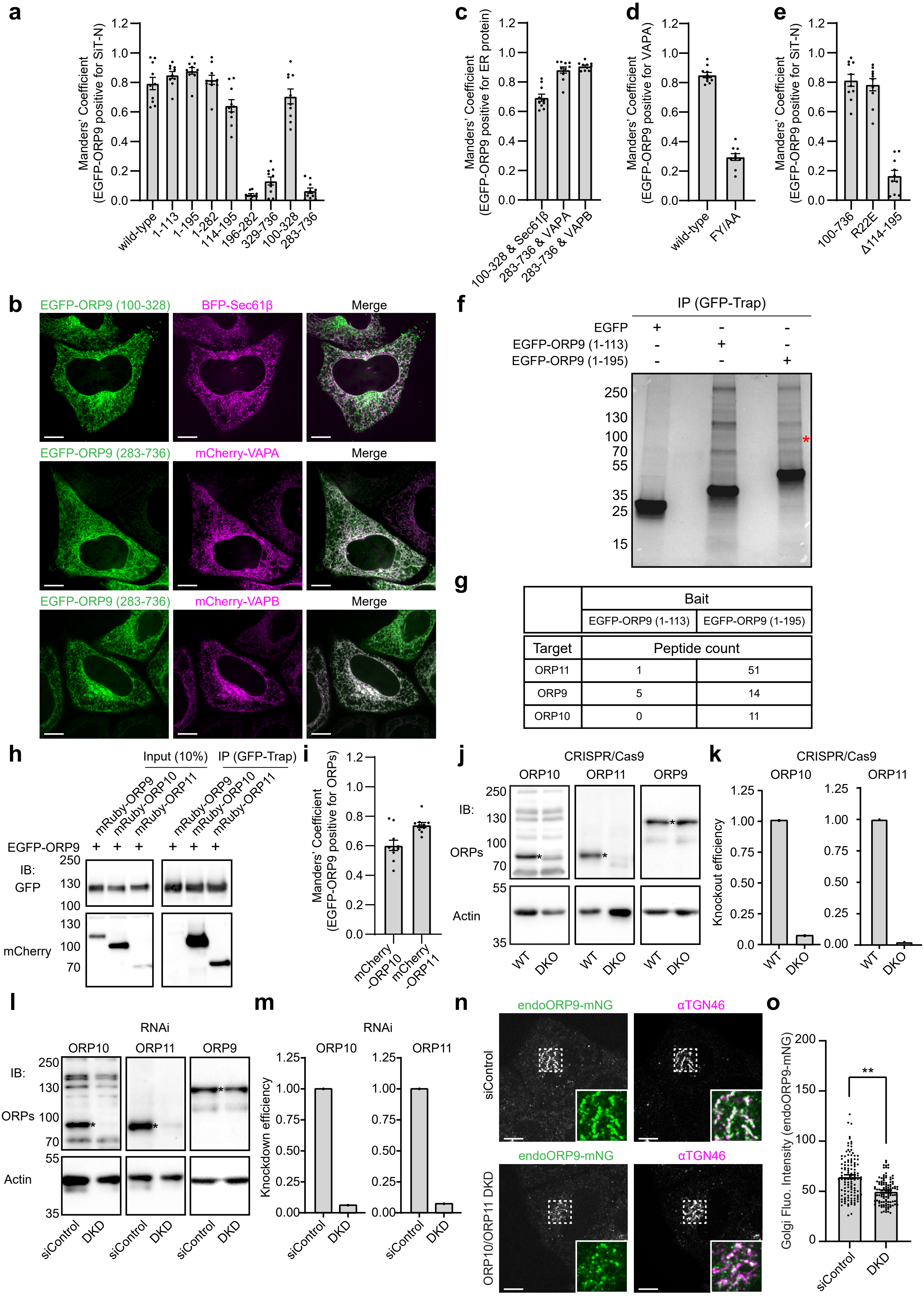

	Bait	
	EGFP-ORP9 (1-113)	EGFP-ORP9 (1-195)
Target	Peptide count	
ORP11	1	51
ORP9	5	14
ORP10	0	11

Supplementary Figure 2. ORP9 localizes to the TGN via its N-terminal PH domain and tandem α-helices. a, c-e. Quantifications of the association of indicated versions of EGFP-ORP9 with indicated proteins as shown in **Figure 2c, Supplementary Figure 2b and Figure 2d, e,** respectively (mean ± SEM, n = 10 cells; Manders' coefficient). **b.** Confocal images of live HeLa cells expressing indicated constructs. Scale bars, 10 μm. **f.** Anti-GFP immunoprecipitates (IP) from the lysates of HEK293T cells expressing indicated constructs were analyzed by SDS-PAGE and stained by colloidal blue. A red asterisk indicates a specific band for EGFP-ORP9 (1-195). **g.** A table showing peptide counts of indicated proteins, as assessed by mass spectrometry, in SDS-PAGE gels cut from regions around the red asterisk as shown in (**f**). **h.** Lysates from HeLa cells expressing the indicated constructs were subjected to anti-GFP IP and then processed for SDS-PAGE and immunoblotting (IB) with indicated antibodies. Inputs are 10% of the total cell lysates. **i.** Quantification of the association of EGFP-ORP9 with mCherry-ORP10 or mCherry-ORP11 as shown in **Figure 2f, g** (mean ± SEM, n = 10 cells; Manders' coefficient). **j.** Lysates of WT and ORP10/ORP11 DKO HeLa cells were processed by SDS–PAGE and IB with indicated antibodies. Asterisks indicate bands corresponding to ORP10, ORP11, or endoORP9-mNG. **k.** Quantification of the signals of ORP10 and ORP11, as shown in (**j**). **l.** Lysates of HeLa cells treated with either control siRNAs (siControl) or siRNAs against ORP10 and ORP11 (DKD) for 96 hrs were processed by SDS–PAGE and IB with indicated antibodies. Asterisks indicate bands corresponding to ORP10, ORP11, or endoORP9-mNeonGreen. **m.** Quantification of the signals of ORP10 and ORP11, as shown in (**l**). **n.** Confocal images of fixed HeLa cells expressing endoORP9-mNG that were treated with indicated siRNAs for 96 hrs. Cells were immunolabeled with antibodies against TGN46. Insets show the regions as indicated by white dashed boxes. Scale bars, 10 μm. **o.** Quantification of endoORP9-mNG fluorescence signals at the Golgi as shown in (**n**) [mean ± SEM, n = 119 cells (siControl), n= 109 cells (DKD); data are pooled from two independent experiments; two-tailed unpaired Student's t-test, **P < 0.0001]. Source data are provided as a Source Data file.

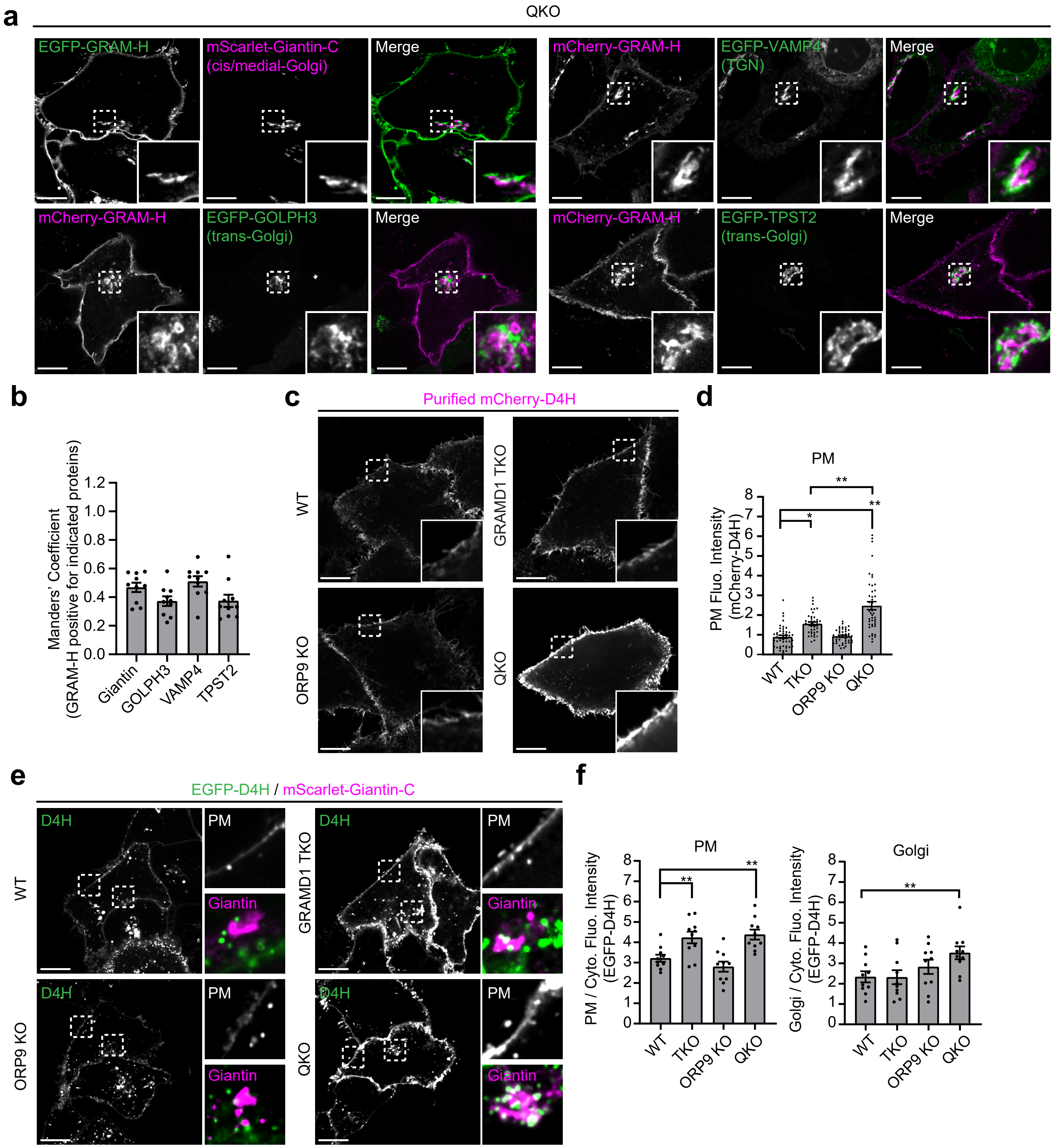

Supplementary Figure 3. Depletion of ORP9 causes accumulation of accessible cholesterol in the Golgi, which is further enhanced by the additional depletion of GRAMD1s with major accumulation in the PM. a. Confocal images of live QKO HeLa cells expressing EGFP-GRAM-H or mCherry-GRAM-H (accessible cholesterol biosensor) together with mScarlet or EGFP tagged Golgi proteins as indicated. Insets show at higher magnification the regions around the Golgi indicated by white dashed boxes. Scale bars, 10 μm. **b.** Quantification of the association EGFP-GRAM-H or mCherry-GRAM-H with indicated Golgi proteins as shown in (**a**) (mean ± SEM, n = 10 cells; Manders' coefficient). **c.** Confocal images of live WT, ORP9 KO, GRAMD1 TKO, and QKO HeLa cells that were stained with recombinant EGFP-D4H proteins (accessible cholesterol biosensor) (10 μg/ml) for 15 min at room temperature. Insets show at higher magnification the regions around the PM indicated by white dashed boxes. Scale bars, 10 μm. **d.** Quantification of PM signals of EGFP-D4H as shown in (**c**) [mean ± SEM, n = 48 cells (WT), n = 42 cells (GRAMD1 TKO), n = 45 cells (ORP9 KO), n = 45 (QKO); data are pooled from two experiments; Tukey's multiple comparisons test, *P = 0.0015, **P < 0.0001]. **e.** Confocal images of live WT, GRAMD1 TKO, ORP9 KO, and QKO HeLa cells expressing EGFP-D4H and mScarlet-Giantin-C. Insets show at higher magnification the regions around the plasma membrane (PM) and the Golgi as indicated by white dashed boxes (for the Golgi; green: EGFP-D4H; magenta: mScarlet-Giantin-C). Scale bars, 10 μm. **f.** Quantification of the ratio of PM signals (left) and Golgi signals (right) to the cytosolic signals of EGFP-D4H as shown in (**e**) (mean ± SEM, n = 10 cells for each condition; data are pooled from one experiment; Dunnett's multiple comparisons test, **P < 0.0001). Source data are provided as a Source Data file.

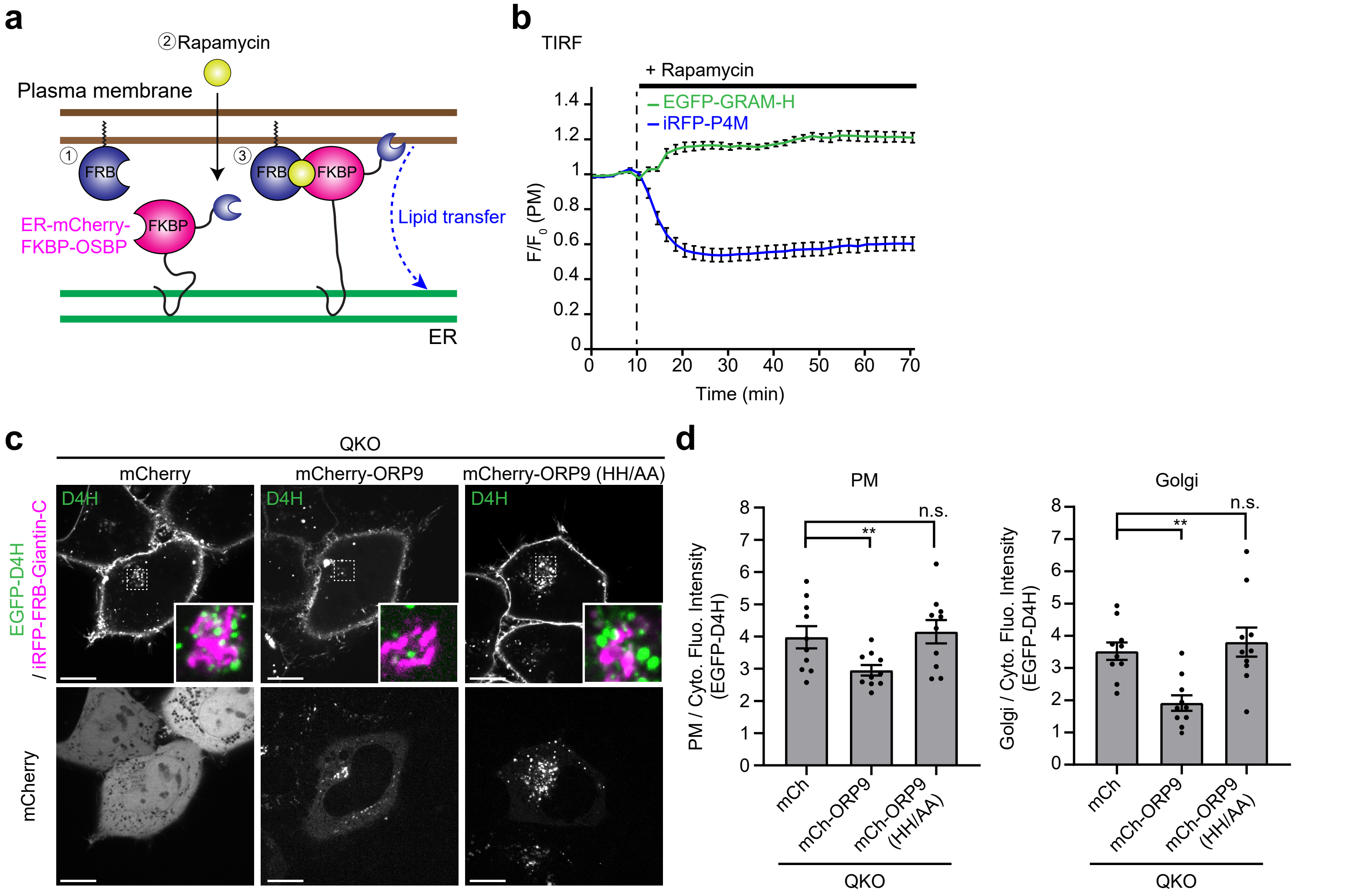

Supplementary Figure 4. The ORD of ORP9 must extract PI4P to maintain proper distribution of cellular cholesterol. a. Schematic representation of the rapamycin-induced recruitment strategy used for the recruitment of ER-anchored ORD of OSBP (ER-mCherry-FKBP-OSBP) to the PM. ER-mCherry-FKBP-OSBP was rapidly recruited to the PM by rapamycin-induced dimerization of FRB and FKBP. ER-mCherry-FKBP-OSBP was expressed in QKO HeLa cells together with a tagBFP-tagged FRB module that is targeted to the PM (PM-FRB-tagBFP). **b.** Time course of normalized signals of EGFP-GRAM-H (accessible cholesterol biosensor) and iRFP-P4M (PI4P biosensor) in response to rapamycin, as assessed by TIRF microscopy of QKO HeLa cells expressing ER-mCherry-FKBP-OSBP and PM-FRB-tagBFP together with EGFP-GRAM-H and iRFP-P4M. Rapamycin addition (200 nM) is indicated (mean ± SEM, n = 40 cells; data are pooled from three independent experiments). **c.** Confocal images of live QKO HeLa cells expressing EGFP-D4H (accessible cholesterol biosensor) together with either mCherry control, mCherry-ORP9, or mCherry-ORP9 carrying PI4P binding-deficient ORD [mCherry-ORP9 (HH/AA)]. Insets show at higher magnification the regions around the Golgi as indicated by white dashed boxes (green: EGFP-D4H; magenta: iRFP-FRB-Giantin-C). Scale bars, 10 μm. **d.** Quantification of the ratio of PM signals (left) and Golgi signals (right) to the cytosolic signals of EGFP-D4H, as shown in (**c**) [mean ± SEM, n = 10 cells; data are pooled from one experiment); Dunnett's multiple comparisons test, **P < 0.0001. n.s. denotes not significant]. Source data are provided as a Source Data file.

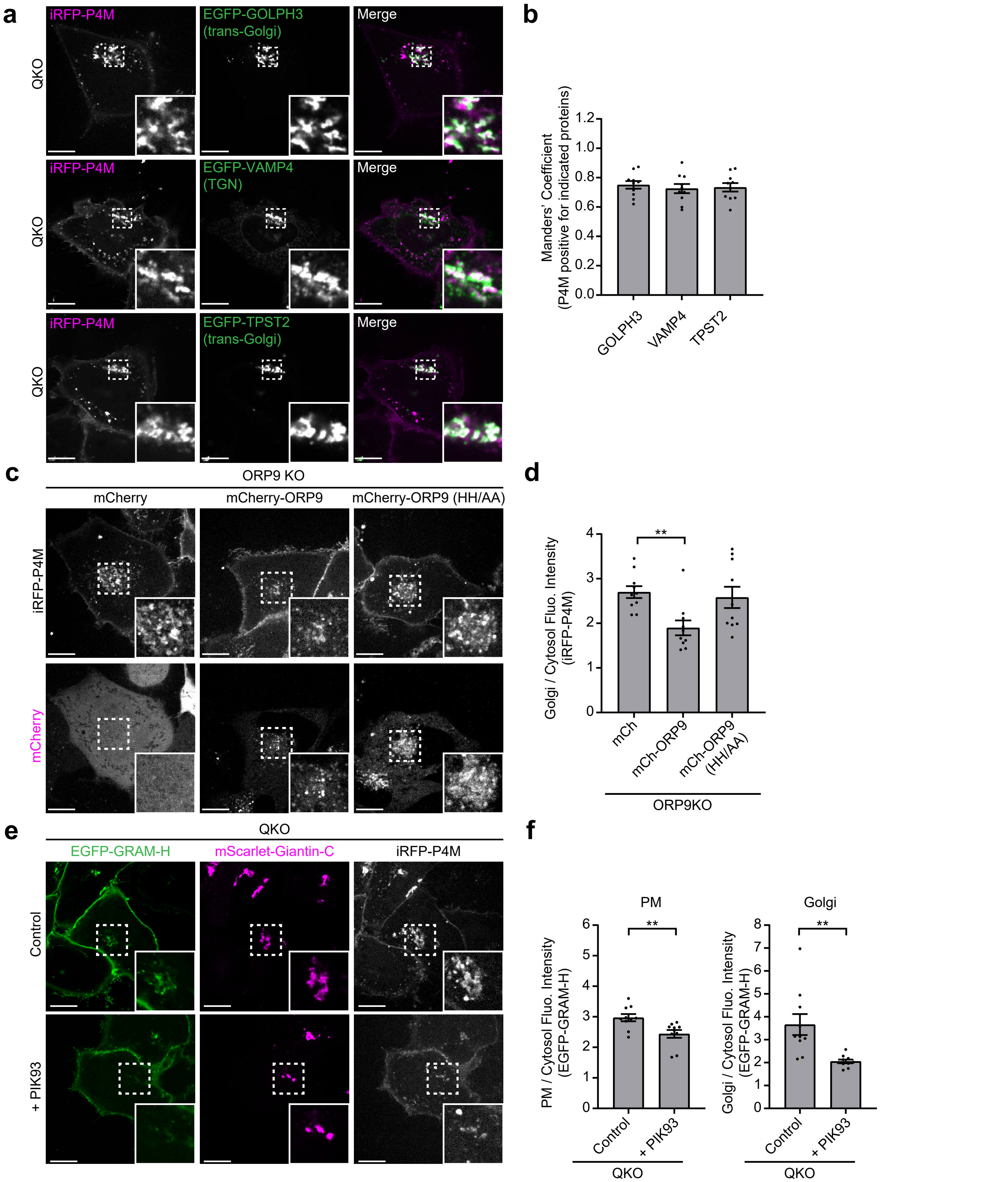

Supplementary Figure 5. ORP9 extraction of PI4P from the TGN helps maintain levels of accessible cholesterol in the Golgi. **a.** Confocal images of live QKO HeLa cells expressing iRFP-P4M together with EGFP-tagged Golgi proteins as indicated. Insets show at higher magnification the regions around the Golgi indicated by white dashed boxes. Scale bars, 10 μm. **b.** Quantification of the association of iRFP-P4M with indicated Golgi proteins as shown in (**a**) (mean ± SEM, n = 10 cells; Manders' coefficient). **c.** Confocal images of live ORP9 KO HeLa cells expressing iRFP-P4M together with either mCherry control, mCherry-ORP9, or mCherry-ORP9 carrying PI4P binding-deficient ORD [mCherry-ORP9 (HH/AA)]. Insets show at higher magnification the regions around the Golgi as indicated by white dashed boxes. Scale bars, 10 μm. **d.** Quantification of the ratio of Golgi signals to the cytosolic signals of mCherry-P4M, as shown in (**c**) (mean ± SEM, n = 10 cells for each condition; data are pooled from one experiment; Dunnett's multiple comparisons test, **P < 0.0001). **e.** Confocal images of live QKO HeLa cells expressing EGFP-GRAM-H together with mScarlet-Giantin-C and iRFP-P4M that were treated with or without PIK93 (250 nM for 1h; PI4KIIIβ inhibitor). Insets show at higher magnification the regions around the Golgi as indicated by white dashed boxes. Scale bars, 10 μm. **f.** Quantification of the ratio of PM signals (left) and Golgi signals (right) to the cytosolic signals of EGFP-GRAM-H, as shown in (**e**) (mean ± SEM, n = 10 cells; data are pooled from one experiment; two-tailed unpaired Student's t-test, **P < 0.0001). Source data are provided as a Source Data file.

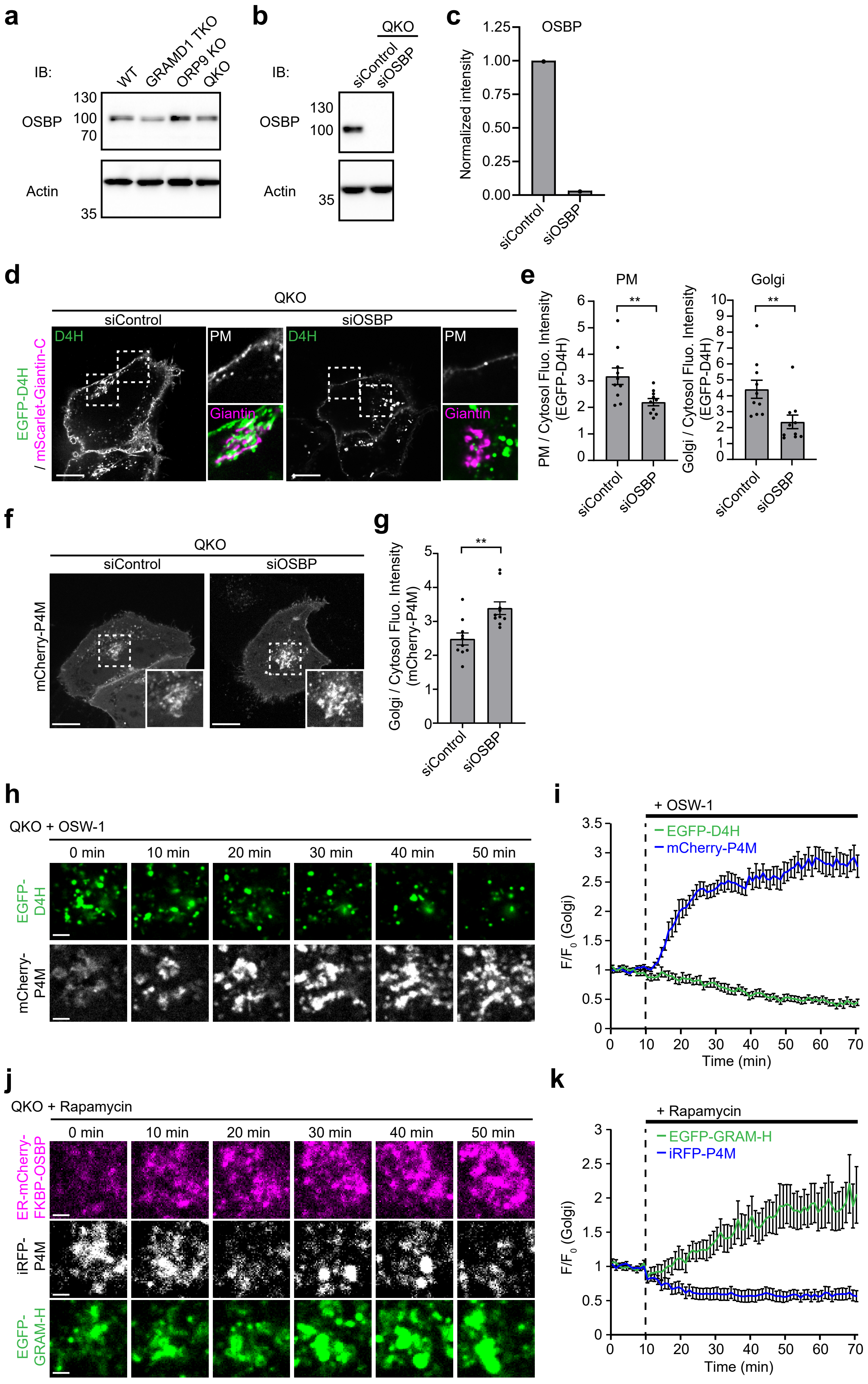

a
IB:
WT
GRAMD1 TKO
ORP9 KO
QKO
OSBP
130
100
70
Actin
35
b
QKO
IB:
siControl
siOSBP
OSBP
130
100
Actin
35
c
OSBP
Normalized intensity
1.25
1.00
0.75
0.50
0.25
0.00
siControl
siOSBP
d
QKO
siControl
siOSBP
D4H
PM
Giantin
EGFP-D4H / mScarlet-Giantin-C
e
PM
Golgi
PM / Cytosol Fluo. Intensity (EGFP-D4H)
Golgi / Cytosol Fluo. Intensity (EGFP-D4H)
**
f
QKO
siControl
siOSBP
mCherry-P4M
g
Golgi / Cytosol Fluo. Intensity (mCherry-P4M)
**
h
QKO + OSW-1
0 min
10 min
20 min
30 min
40 min
50 min
EGFP-D4H
mCherry-P4M
i
+ OSW-1
EGFP-D4H
mCherry-P4M
F/F0 (Golgi)
Time (min)
j
QKO + Rapamycin
ER-mCherry-FKBP-OSBP
iRFP-P4M
EGFP-GRAM-H
k
+ Rapamycin
EGFP-GRAM-H
iRFP-P4M
F/F0 (Golgi)
Time (min)

Supplementary Figure 6. Accumulation of TGN PI4P in QKO cells is associated with hyperactivation of OSBP-mediated cholesterol transport to the Golgi. a. Lysates from indicated HeLa cells were processed by SDS-PAGE and immunoblotting (IB) with indicated antibodies. **b.** Lysates of QKO HeLa cells treated with indicated siRNA for 72 hrs were processed by SDS–PAGE and IB with indicated antibodies. **c.** Quantification of the signals of OSBP normalized with those of actin, as shown in (**b**). **d.** Confocal images of live QKO HeLa cells expressing indicated constructs that were treated with indicated siRNAs for 72 hrs. Insets show the regions as indicated by white dashed boxes (for the Golgi, green: EGFP-D4H; magenta: mScarlet-Giantin-C). Scale bars, 10 μm. **e.** Quantification of the ratio of PM signals (left) and Golgi signals (right) to the cytosolic signals of EGFP-D4H, as shown in (**d**) (mean ± SEM, n = 10 cells; data are pooled from one experiment; two-tailed unpaired Student's t-test, $^{**}P < 0.0001$). **f.** Confocal images of live QKO HeLa cells expressing mCherry-P4M that were treated with indicated siRNAs for 72 hrs. Insets show the regions as indicated by white dashed boxes. Scale bars, 10 μm. **g.** Quantification of the ratio of Golgi signals to the cytosolic signals of mCherry-P4M, as shown in (**f**) (mean ± SEM, n = 10 cells for each condition; data are pooled from one experiment; two-tailed unpaired Student's t-test, $^{**}P = 0.0025$). **h.** Confocal images of a live QKO HeLa cell expressing EGFP-D4H and iRFP-P4M that were treated with OSW-1 (20 nM) as indicated. Scale bars, 2 μm. **i.** Time course of normalized signals of EGFP-D4H and iRFP-P4M at the regions around the TGN as shown in (**h**) (mean ± SEM, n = 12 cells for each condition; data are pooled from two independent experiments). **j.** Confocal images of a live QKO HeLa cell expressing ER-mCherry-FKBP-OSBP and tagBFP-TGN38-FRB together with EGFP-GRAM-H and iRFP-P4M that were treated with rapamycin (200 nM) as indicated. Scale bars, 2 μm **k.** Time course of normalized signals of EGFP-GRAM-H and iRFP-P4M at the regions around the TGN as shown in (**j**) (mean ± SEM, n = 16 cells; data are pooled from four independent experiments). Source data are provided as a Source Data file.

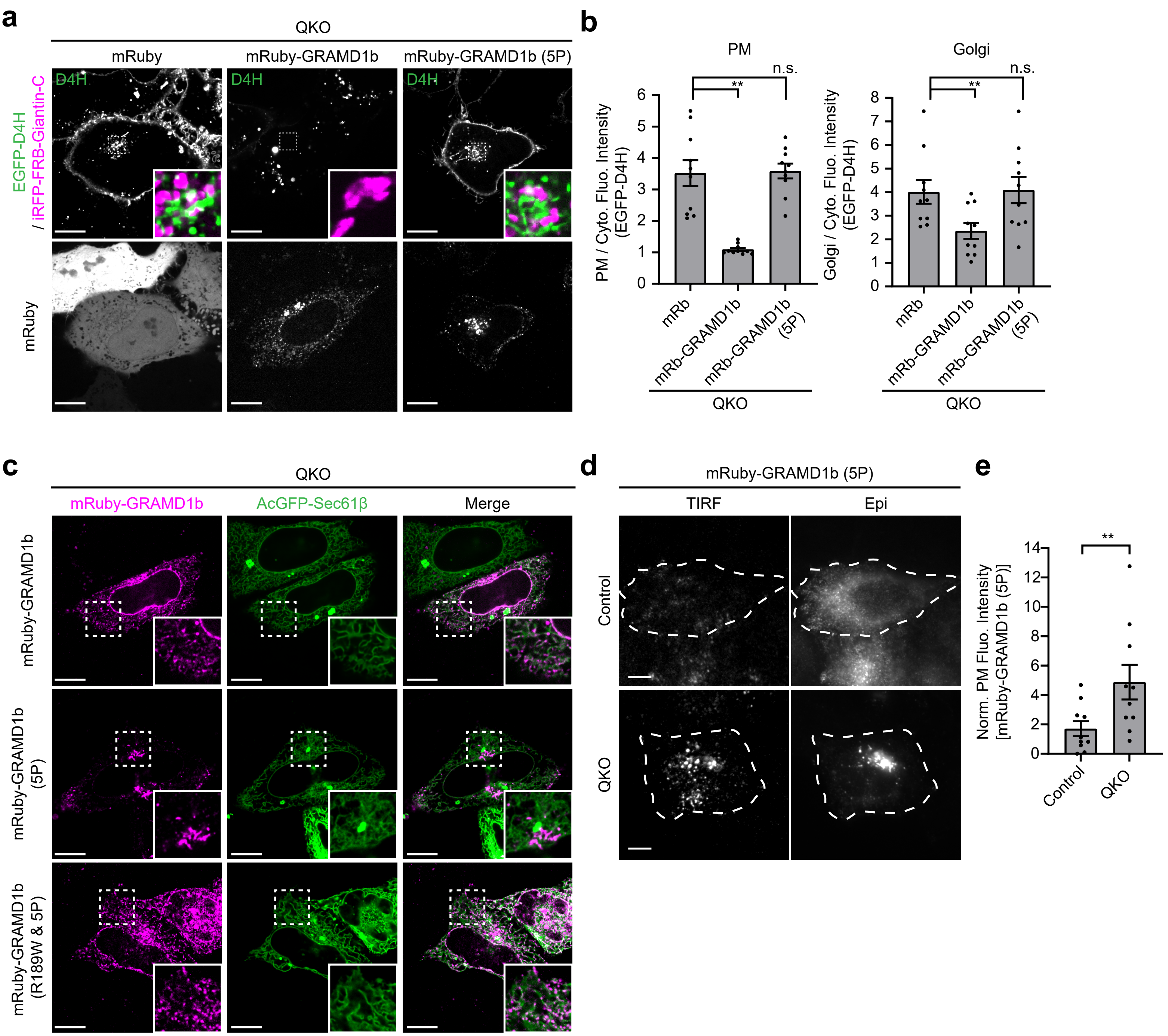

Supplementary Figure 7. GRAMD1b acts at ER-TGN contact sites to remove excess cholesterol from the Golgi. a. Confocal images of live QKO HeLa cells expressing either mRuby control, mRuby-tagged GRAMD1b (mRuby-GRAMD1b), or mRuby-GRAMD1b carrying cholesterol binding-deficient StART-like domain [mRuby-GRAMD1b (5P)] together with EGFP-D4H (accessible cholesterol biosensor). Insets show at higher magnification the regions around the Golgi as indicated by white dashed boxes (green: EGFP-D4H; magenta: mScarlet-Giantin-C). Scale bars, 10 μm. **b.** Quantifications of the ratio of PM signals (left) and Golgi signals (right) to the cytosolic signals of EGFP-D4H, as shown in (**a**) (mean ± SEM, n = 10 cells for each condition; data are pooled from one experiment; Dunnett's multiple comparisons test, $^{**}P < 0.0001$, n.s. denotes not significant). **c.** Confocal images of live QKO HeLa cells expressing either mRuby-GRAMD1b, mRuby-GRAMD1b (5P), or mRuby-GRAMD1b carrying cholesterol sensing-deficient GRAM domain and 5P mutations [mRuby-GRAMD1b (R189W & 5P)] together with a ER marker, AcGFP-tagged Sec61β (AcGFP-Sec61β). Scale bars, 10 μm. **d.** Total internal reflection fluorescence (TIRF) images and epifluorescence (Epi) images of live WT control and QKO HeLa cells expressing mRuby-GRAMD1b (5P). White dashed lines indicate a region covering a single cell. **e.** Quantifications of PM signals (as assessed by TIRF images) that were normalized by total signals (as assessed by Epi) of mRuby-GRAMD1b (5P), as shown in (**d**) (mean ± SEM, n = 10 cells for each condition; data are pooled from one experiment; two-tailed unpaired Student's t-test, $^{**}P < 0.0001$). Source data are provided as a Source Data file.

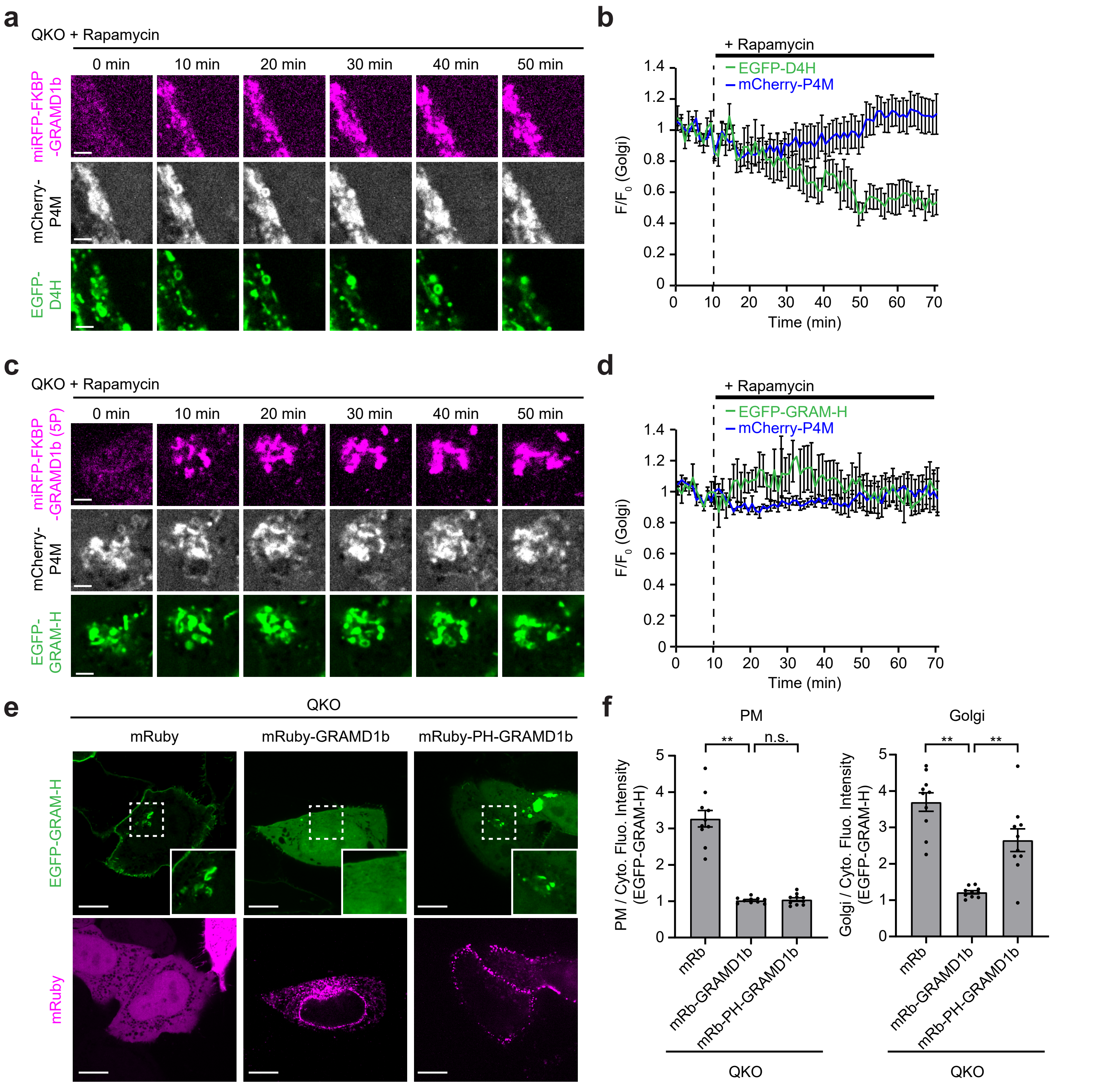

Supplementary Figure 8. GRAMD1b that cannot transport cholesterol (5P) accumulates at the Golgi and PM in QKO cells. **a.** Representative confocal images of a live QKO HeLa cell expressing miRFP-FKBP-GRAMD1b and tagBFP-TGN38-FRB together with EGFP-D4H (accessible cholesterol biosensor) and mCherry-P4M (PI4P biosensor) that were treated with rapamycin (200 nM) for the indicated minutes. Scale bars, 2 μm. **b.** Time course of normalized signals of EGFP-D4H and mCherry-P4M at the regions around the TGN in response to rapamycin, as assessed by confocal microscopy as shown in (**a**) (mean ± SEM, n = 11 cells; data are pooled from three independent experiments). **c.** Representative confocal images of a QKO HeLa cell expressing miRFP-FKBP-GRAMD1b (5P), which is carrying cholesterol binding-deficient StART like domain, and tagBFP-TGN38-FRB together with EGFP-GRAM-H (accessible cholesterol biosensor) and mCherry-P4M that were treated with rapamycin (200 nM) for the indicated minutes. Scale bars, 2 μm. **d.** Time course of normalized signals of EGFP-GRAM-H and mCherry-P4M at the regions around the TGN in response to rapamycin, as assessed by confocal microscopy as shown in (**c**) (mean ± SEM, n = 8 cells; data are pooled from two independent experiments). **e.** Confocal images of live QKO HeLa cells expressing either mRuby control, mRuby-GRAMD1b or mRuby-tagged PH-GRAMD1b, whose GRAM domain was replaced by a PM-interacting module (i.e., the PH domain of PLCδ1) (mRuby-PH-GRAMD1b), together with EGFP-GRAM-H. Insets show at higher magnification the regions around the Golgi indicated by white dashed boxes. Scale bars, 10 μm. **f.** Quantifications of the ratio of PM signals (left) and Golgi signals (right) to the cytosolic signals of EGFP-GRAM-H, as shown in (**e**) (mean ± SEM, n = 10 cells for each condition; data are pooled from one experiment; Dunnett's multiple comparisons test, **P < 0.0001, n.s. denotes not significant). Source data are provided as a Source Data file.

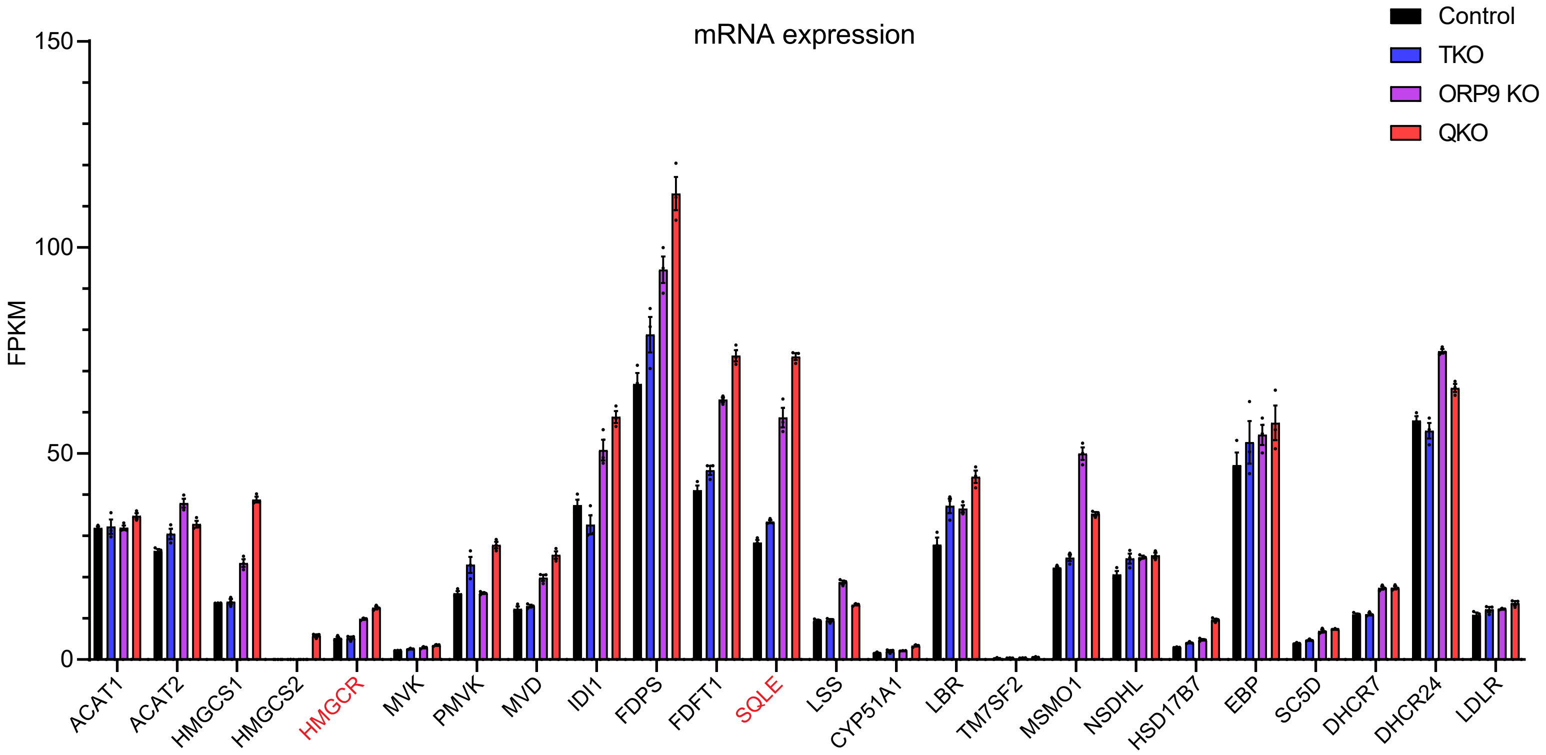

Supplementary Figure 9. QKO cells exhibit dysregulated SREBP-2 signalling and increased cholesterol production. mRNA expression profile of genes involved in mevalonate pathway. RNA was extracted from cells that had been cultured in the medium supplemented with 10% FBS, and the abundance of mRNA was analyzed for each condition via massively parallel sequencing. Fragments per kilo bases per million reads (FPKM) values of each gene were plotted (mean ± SEM, n = 3 independent experiments). Genes shown in red are rate limiting enzymes in the mevalonate pathway. Source data are provided as a Source Data file.

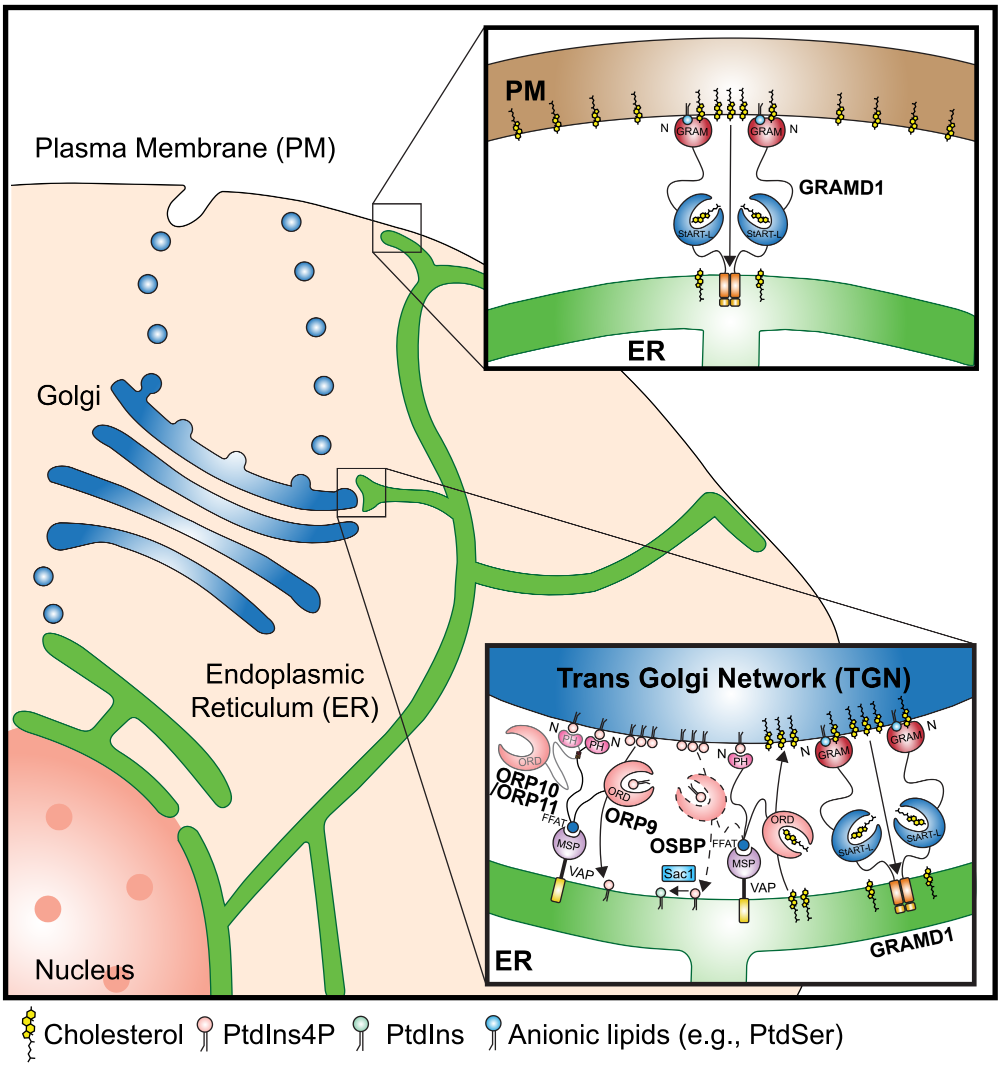

Supplementary Figure 10. Regulation of cellular cholesterol distribution via non-vesicular lipid transport at membrane contact sites. Graphical abstract showing the importance of non-vesicular lipid transport regulated by lipid transfer proteins at membrane contact sites for the maintenance of cellular cholesterol distribution. ORP9 localizes to the TGN via its tandem α-helices, which interact with ORP10 and ORP11. ORP9 extracts PI4P from the TGN and contributes to the suppression of OSBP-mediated PI4P-driven cholesterol transport to the Golgi, whereas GRAMD1s sense transient expansions of the accessible pool of cholesterol in the Golgi as well as in the PM (and possibly in other cellular organelles/membranes) and transfer it to the ER to prevent its build-up.

Supplementary Table 1

REAGENT or RESOURCE	SOURCE	IDENTIFIER
**Antibodies**		
Anti-ORP9 (1:100 dilution for IF)	Sigma-Aldrich/Merck	RRID:AB_1854817
Anti-ORP9 (1:1,000 dilution for WB)	Abcam	ab151691
Anti-OSBP(1:100 dilution for IF; 1:1,000 dilution for WB)	Sigma-Aldrich/Merck	RRID:AB_2676401
Anti-TGN46 (1:100 dilution for IF)	BIO RAD	RRID:AB_2203291
Anti-TGN46 (1:100 dilution for IF)	Proteintech	RRID:AB_10597396
Anti-GM130 (1:100 dilution for IF)	BD Biosciences	RRID:AB_398141
Anti-Actin (1:1,000 dilution for WB)	EMD Millipore	RRID:AB_2223041
Anti-SREBP-2 (1:500 dilution for WB)	Santa Cruz Biotechnology	RRID: AB_2194250
Anti-mNeonGreen (1:1,000 dilution for WB)	Chromotek	RRID:AB_2827566
Anti-mCherry (1:1,000 dilution for WB)	Proteintech	RRID:AB_2876881
Anti-ORP10 (1:1,000 dilution for WB)	Proteintech	RRID:AB_2158356
Anti-ORP11 (1:1,000 dilution for WB)	Origene	RRID:AB_11124393
Goat Anti-Rabbit IgG (H+L)-HRP Conjugate (1:5,000 dilution for WB)	Bio Rad	RRID:AB_11125142
Goat Anti-Mouse IgG (H+L)-HRP Conjugate (1:5,000 dilution for WB)	Bio Rad	RRID:AB_11125547
Donkey anti-Sheep IgG (H+L) Cross-Adsorbed Secondary Antibody, Alexa Fluor™ 594 (1:200 dilution for IF)	Thermo Fisher Scientific	RRID:AB_10562537
**Chemicals, Peptides, and Recombinant Proteins**		
OSW-1	Cayman Chemical	30310
PIK93	Sigma-Aldrich/Merck	SML0546
Rapamycin	Sigma-Aldrich/Merck	R0395
COLLOIDAL BLUE STAINING KIT	Thermo Fisher Scientific	LC6025
Benzonase® Nuclease	Santa Cruz Biotechonology	sc-202391
Puromycin	Stemcell	73342
Opti-MEM™ I Reduced Serum Medium	Thermo Fisher Scientific	31985070
Lipofectamine 2000 Reagent	Thermo Fisher Scientific	11668-019
Lipofectamine RNAiMAX	Thermo Fisher Scientific	13778150
Fetal Bovine Serum	Gibco	10270-106
DMEM(4.5g/l Glucose) with L-Gln, without Sodium Pyruvate	Nacalai Tesque, Japan	08459-35
Amphotericin B	Sigma-Aldrich/Merck	A4888
Alt-R™ S.p. Cas9 Nuclease V3	IDT	1081058
**Critical Commercial Assays**		
Q5 Site-Directed Mutagenesis Kit	NEB	E0552S
NEBuilder HiFi DNA Assembly Cloning Kit	NEB	E5520S
BCA Protein Assay Kit	NEB	23225
CellTiter-Glo® Luminescent Cell Viability Assay Kit.	Promega	G7570
AmplexRed cholesterol assay kit	Thermo Fisher Scientific	A12216
**Experimental Models: Cell Lines**		

HeLa M	Others	Gift from Pietro De Camilli
HeLa GRAMD1 TKO	Naito et al (2019)	
HeLa ORP9 KO	This paper	
HeLa GRAMD1 TKO-ORP9 KO (QKO)	This paper	
QKO expressing EGFP-GRAM-H	This paper	
QKO expressing EGFP-GRAM-H and ER-mCherry-FKBP-ORP9	This paper	
QKO expressing EGFP-GRAM-H and ER-mCherry-FKBP-ORP9 (HH/AA)	This paper	
QKO expressing GRAM-H and ER-mCherry-FKBP-OSBP	This paper	
HeLa expressing endoORP9-mNG and mSc-endoOSBP	This paper	DKI HeLa cells
DKI HeLa ORP10/ORP11 DKO	This paper	
HEK293T	Others	Gift from Nguan Soon Tan
**Oligonucleotides**		
CACCGGTGCTGCCAAGCATTAGGT	This paper	ORP9_ORANGE_4_CT_S
AAACACCTAATGCTTGGCAGCACC	This paper	ORP9_ORANGE_4_CT_AS
TCTAGAAGCTTCCAACCTAATGCTTGGCAGCACCGGTTCTGGATCGGGTGTGAGCAAGGGCGAGGAGGATAAC	This paper	HindIII_ORP9_ORANGE_4_mNG_NS
GGATCCTCGAGGGTGCTGCCAAGCATTAGGTTGGCTACTTGTACAGCTCGTCCATGCCCATC	This paper	XhoI_ORP9_ORANGE_4_mNG_stop_CAS
CACCGATTGCAGCACTTGGCGGCGG	This paper	OSBP_ORANGE_gRNA_2_NT_S
AAACCCGCCGCCAAGTGCTGCAATC	This paper	OSBP_ORANGE_gRNA_2_NT_AS
TCTAGAAGCTTCCGCCGCCGCCAAGTGCTGCAATGCGGTTCTGGATCGGGTGTGAGCAAGGGCGAGGCAGTG	This paper	HindIII_pORANGE_mScarlet-OSBP_site_2_S
AGGATCCTCGAGATTGCAGCACTTGGCGGCGGCGGGCGCTACCTGAACCCTTGTACAGCTCGTCCATGCCGC	This paper	XhoI_pORANGE_mScarlet-OSBP_site_2_AS
GAGGGTCCGGACTCAGATCTCGAG	This paper	5'_MCS-XhoI_S
GGTGGATCCCGGGCCCGCGGTACC	This paper	3'_MCS_KpnI_AS
ACTCAGATCTCGAGGAATGAACGGAGAGGAAGAATTCTTTGATGC	This paper	5'_XhoI_ORP2_NS
GGGCCCGCGGTACCTCAGTAGATATCTGGGCAGTCGGAGAAATTC	This paper	3'_KpnI_stop_ORP2_CAS
ACTCAGATCTCGAGCTATGATGAGTGATGAGAAGAACCTTGGTG	This paper	5'_XhoI_ORP3_NS
CGGGCCCGCGGTACCTCACCATAAGACAGGATGGTCCAGTTTGG	This paper	3'_KpnI_Stop_ORP3_CAS
AGCTCAAGCTTCGAATTCGATGGACTTCCAAGAGAGGGACCCGCC	This paper	5'_EcoRI_ORP7_NS
TATCTAGATCCGGTGGATCCCGGGCTACCAGAGCACGGCCCCATC	This paper	3'_SmaI_stop_ORP7_CAS
TCTCGAGCTCAAGCTTCGATGGAGAGGGCAGTCCAGGGCACAGAC	This paper	5'_HindIII_ORP10_NS
CGGGCCCGCGGTACCTCAGTGTGCTTTCCAGAGGGGATTGAAG	This paper	3'_KpnI_stop_ORP10_CAS

ACTCAGATCTCGAGCTATGCAGGGGGGTGAACCAGTGTCCAC	This paper	5'_XhoI_ORP11_NS
CGGGCCCGCGGTACCTCACTCTGCTGGTTGTGTTGTTGGAATTAT	This paper	3'_KpnI_stop_ORP11_CAS
TCAGATCTCGAGCTATGGCGTCCATCATGGAAGGGCCGCTGAG	This paper	XhoI_ORP9_1_forward
GCCCGCGGTACCCTAAACAAATCCTGAATCCAAACCTTGAAGCTG	This paper	KpnI_ORP9_113_reverse
GCCCGCGGTACCCTAACTTATCATTCCATCTGCGTGCTTCTCCG	This paper	KpnI_ORP9_195_reverse
GCCCGCGGTACCCTATGTATTTGGAGACAAGTTCACGTGGCTTG	This paper	KpnI_ORP9_282_reverse
TCAGATCTCGAGCTACTATTAATCCCGTAGATGCAATATATCAAC	This paper	XhoI_ORP9_196_forward
CTCAGATCTCGAGCTCGACATACTCTCCAGCTTCAAGGTTTG	This paper	XhoI_ORP9L_100aa_S
CGCGGTACCCTAATTTCCCGAGCTGCTGTGTGTCAG	This paper	KpnI_ORP9L_328_AS
TCAGATCTCGAGCTGTCCCAGAGTTCTCTTACTCCAGCAGTGAAG	This paper	XhoI_ORP9_283_forward
GTGGATCCCGGGCTAATGCTTGGCAGCACCAAGACGTTTCAGTAA	This paper	XmaI_ORP9_736_reverse
TCAGATCTCGAGCTAGTCTAAAACGCCCAGATACCACAGAATCAC	This paper	XhoI_ORP9_329_forward
CTCAGATCTCGAGCTCGACATACTCTCCAGCTTCAAGGTTTG	This paper	XhoI_ORP9L_100aa_S
GAGTGGTTCGTGCTGGACTACAATGCAG	This paper	ORP9_R22E_S
GTACTGCCAGCCCTTCATCACGTTAG	This paper	ORP9_R22E_AS
TGGTAGTGCTGGTGGTAGTGGTACCAGTCTAAAACGCCCAGATACCACAG	This paper	link-KpnI-ORP9_329aa_S_hifi
ATCAGTTATCTAGATCCGGTGGATCCCTAATGCTTGGCAGCACCAAGAC	This paper	BamHI-ORP9L_CAS_Hifi
GGTTTAGTGAACCGTCAGATCCGCTAGCGCCACCATGAGCGGCGCCCGGGGCGAG	This paper	NheI-kozak-E-Syt2_S_Hifi
CCTCGCCCTTGCTCACCATGGTGGCTTCAGTGTCTGGAAAATGAACCCAGGC	This paper	E-Syt2_mCherry_AS_Hifi
TCGAGAATTCCTAATGCTTGGCAGCACCAAGAC	This paper	EcoRI-stop-ORP9L_CAS
TCCACCCATTTCAGCCTTTTATGCTGAG	This paper	ORP9L_HAHA_S
GCAGCGGAAACCTGCTCAGCCACAAATG	This paper	ORP9L_HAHA_AS
CACCGAAGGATCCCAAGGATCGAA	This paper	ORP9-sgRNA#1_S
AAACTTCGATCCTTGGGATCCTTC	This paper	ORP9-sgRNA#1_AS
CACCGTCGCCCAAAATGGGATTGTA	This paper	ORP9-sgRNA#2_S
AAACTACAATCCCATTTTGGGCGAC	This paper	ORP9-sgRNA#2_AS
CTGTAAGCTAACTCCTGGTCCTAG	This paper	ORP9_Exon16_GT_S2
CACTACATTCAAACTATGCTGCTC	This paper	ORP9_Exon16_GT_AS2
GCAGTTGCTCCAACGTTTATTCTTGAAAGAAGATCTCTTTTAG	This paper	ORP9_AAA_mut_S
CTTAGTAGCATCCATTCCAAGTCTAACCTGCGAC	This paper	ORP9_AAA_mut_AS
GGACTCAGATCTCACGGGCTCCAGGATGACCC	This paper	BglII-PHplcd-S

CTCGAGATCTCTTCTGCCGCTGGTCCATGGAG	This paper	BglII-PHplcd-AS
GATCCGCTAGCGCTACCGGTCGCCACCATGGTGAG CAAGGGCGAG	This paper	NheI-mCherry-NS
CCGCCTCCACCGGTCTTTTCAAGGAGAGCATTCTGC CAGAGCCG	This paper	AgeI_delStop_GRA M1b_AS
GGGCCCGCGGTACCTTAATTGTAGGTTATGGAACTA CCTG	This paper	5’ EcoRI D4_Mam
GGGCCCGCGGTACCTTAATTGTAGGTTATGGAACTA CCTG	This paper	3’ KpnI_D4_Mam_Sto p
GGGACGGGAATTATCAGAGCAAAACAGCACATTAC	This paper	D4 D434S F
AAGTCTTATGGGTCAGGACCTCATTCCCCTC	This paper	D4 D434S R
mA*mC*mA*rArGrUrUrGrArGrGrCrArCrUrCrCrCrCrArGr UrUrUrUrArGrArGrCrUrArGrArArArUrArGrCrArArGrUrUr ArArArArUrArArGrGrCrUrArGrUrCrCrGrUrUrArUrCrArAr CrUrUrGrArArArArArGrUrGrGrCrArCrCrGrArGrUrCrGrGr UrGrCmU*mU*mU*rU	This paper	ORP10-sgRNA#1
mG*mG*mG*rArCrUrCrArArUrGrGrCrGrUrGrCrArCrArGr UrUrUrUrArGrArGrCrUrArGrArArArUrArGrCrArArGrUrUr ArArArArUrArArGrGrCrUrArGrUrCrCrGrUrUrArUrCrArAr CrUrUrGrArArArArArGrUrGrGrCrArCrCrGrArGrUrCrGrGr UrGrCmU*mU*mU*rU	This paper	ORP10-sgRNA#2
mC*mU*mC*rArUrUrUrCrArUrGrArArGrGrCrCrGrUrArGr UrUrUrUrArGrArGrCrUrArGrArArArUrArGrCrArArGrUrUr ArArArArUrArArGrGrCrUrArGrUrCrCrGrUrUrArUrCrArAr CrUrUrGrArArArArArGrUrGrGrCrArCrCrGrArGrUrCrGrGr UrGrCmU*mU*mU*rU	This paper	ORP11-sgRNA#1
mG*mU*mC*rArArArGrArCrUrCrCrCrCrCrGrArUrArArGr UrUrUrUrArGrArGrCrUrArGrArArArUrArGrCrArArGrUrUr ArArArArUrArArGrGrCrUrArGrUrCrCrGrUrUrArUrCrArAr CrUrUrGrArArArArArGrUrGrGrCrArCrCrGrArGrUrCrGrGr UrGrCmU*mU*mU*rU	This paper	ORP11-sgRNA#2
AAGGGGAAAATCAATTTGGACCATTCTGGAGCATAC GTAGCTCAATTTGAGGTCGCATGGGACGAGGTAAGT TATGACAAAGAGGGGAATGAGGTCCTGACCCATAAG ACTTGGGACGGGAATTATCAGGACAAAACAGCACAT TACAGTACCGTAATCCCGCTGGAGGCAAATGCTCGA AACATAAGGAAAAGGCCCGAGAATGCACAGGTCTTG CCTGGAGTGGTGGCGAGACGTGATTTCTGAATTGAC GTCCCCCTCACCAATAACATCAACGCTCTATTTGGG GGACGACTCTCTATCCAGGTAGTTCCATAACCTACAA T TAA	This paper	D4 for mammalian expression

TGGGCGGAGGGTCCGGACTCAGATCTCGAGCTATG<br>AACACAGAAGCGGAGCAACAGCTTCTCCATCACGCC<br>AGAAATGGCAATGCTGAAGAAGTAAGACAACTATTA<br>GAGACCATGGCGAGGAATGAAGTGATTGCTGACATT<br>AATTGCAAAGGAAGAAGTAAGTCTAACTTGGGCTGG<br>ACACCTCTACATCTGGCATGCTATTTTGGACACAGAC<br>AAGTGGTCCAGGATCTGTTGAAGGCTGGTGCAGAAG<br>TGAATGTGTTGAATGACATGGGAGACACGCCGCTTC<br>ATCGAGCTGCCTTTACAGGACGAAAGGAGTTGGTAA<br>TGCTTCTCTTAGAATATAATGCTGATACTACTATTGTT<br>AATGGGAGTGGACAGACAGCAAAAGAAGTTACTCAT<br>GCTGAAGAAATCAGAAGCATGCTTGAAGCTGTAGAA<br>AGGACTCAACAAAGAAAGCTTGAAGAATTACTTTTAG<br>CAGCAGCAAGAGAAGGCAAAACAACAGAACTCACAG<br>CTCTGCTCAACAGGCCCAATCCTCCTGATGTTAACT<br>GTTCGGATCAGTTAGGAAATACACCCTTGCATTGTG<br>CAGCTTACCGGGCCCATAAACAATGTGCCTTAAAGC<br>TTCTAAGAAGTGGAGCAGACCCTAATCTGAAGAACA<br>AAAATGATCAGAAACCTCTTGACCTTGCCCAGGGTG<br>CTGAAATGAAACACATTCTTGTTGGTAATAAGGTCAT<br>CTACAAAGCATTGAAACGATATGAGGGCCCTCTCTG<br>GAAGAGTTCAAGATTTTTTGGCTGGAGATTATTCTGG<br>GTAGTGTTAGAGCATGGAGTCCTTTCATGGTATAGG<br>AAACAGCCTGATGCAGTTCATAATATTTATCGCCAGG<br>GATGCAAACACCTGACTCAAGCAGTATGCACGGTAA<br>AATCCACTGATAGCTGCCTCTTCTTTATTAAATGCTTT<br>GATGACACCATTCATGGCTTCCGGGTTCCTAAGAAT<br>AGCCTTCAGCAGTCAAGAGAGGACTGGCTGGAAGC<br>AATAGAAGAACATTCTGCTTACAGCACTCACTACTGT<br>TCCCAGGACCAGCTGACTGATGAGGAGGAGGAAGA<br>TACGGTTTCTGCTGCAGACCTGAAGAAATCATTAGA<br>GAAAGCACAGTCATGCCAACAGCGACTAGATAGGGA<br>AATTTCCAACTTTCTCAAAATGATTAAGGAGTGTGAC<br>ATGGCTAAAGAAATGCTTCCATCATTTCTTCAGAAAG<br>TTGAAGTTGTCTCAGAAGCTTCTAGAGAAACTTGTGT<br>AGCTTTGACTGATTGCCTTAATCTCTTCACCAAACAA<br>GAAGGGGTGAGGAATTTTAAATTGGAACAAGAGCAA<br>GAAAAAAACAAAATCTTGTCAGAAGCACTGGAGACG<br>CTGGCCACTGAACATCATGAATTAGAGCAGTCTCTG<br>GTGAAAGGCTCTCCACCCGCCAGCATCCTTAGCGAG<br>GACGAGTTCTATGATGCGCTGTCAGATTCCGAGTCC<br>GAAAGG	This paper	XhoI_ORP1L_Frag1

GCTGTCAGATTCCGAGTCCGAAAGGTCCCTGAGTAG ATTGGAAGCAGTGACAGCACGCTCCTTTGAAGAGGA AGGAGAGCATTTGGGCAGTAGAAAACACAGAATGTC CGAAGAAAAAGACTGTGGTGGCGGAGATGCTCTCTC CAATGGCATCAAGAAACACAGAACAAGTTTGCCTTCT CCTATGTTTTCCAGAAATGACTTCAGTATCTGGAGCA TCCTCAGAAAATGTATTGGAATGGAACTATCCAAGAT CACGATGCCAGTTATATTTAATGAGCCTCTGAGCTTC CTACAGCGCCTAACTGAATACATGGAGCATACTTAC CTCATCCACAAGGCCAGTTCACTCTCTGATCCTGTG GAAAGGATGCAGTGTGTAGCTGCGTTTGCTGTATCT GCTGTTGCTTCTCAGTGGGAACGGACTGGAAAACCT TTCAACCCACTGCTGGGAGAGACTTATGAATTAGTG CGAGATGACCTTGGATTTAGACTCATCTCCGAACAG GTCAGCCATCACCCACCAATCAGTGCATTTCATGCT GAAGGATTAAACAATGACTTCATCTTTCATGGCTCTA TCTATCCCAAACTGAAATTCTGGGGGAAGAGTGTAG AAGCAGAACCCAAAGGAACCATCACCTTGGAGCTCC TTGAACACAATGAGGCATATACATGGACAAATCCCAC CTGCTGTGTGCATAATATCATTGTGGGTAAACTGTGG ATCGAACAGTATGGCAATGTGGAAATTATAAACCACA AGACTGGGGACAAATGTGTGTTGAATTTTAAGCCAT GTGGCCTTTTTGGTAAGGAATTACACAAAGTTGAAG GCTACATTCAAGATAAAAGCAAAAAGAAGCTCTGTGC CCTCTATGGGAAGTGGACTGAATGTTTATACAGTGTT GACCCTGCCACGTTTGACGCTTACAAAAAAAATGATA AGAAAAATACAGAAGAGAAGAAGAACAGCAAACAGA TGAGCACCTCTGAGGAGTTGGATGAAATGCCAGTGC CGGATTCTGAAAGTGTATTCATTATCCCTGGAAGCGT TCTTCTATGGCGAATAGCCCCACGGCCTCCAAATTC TGCCCAGATGTATAATTTTACTAGTTTTGCAATGGTTT TGAATGAAGTAGACAAAGACATGGAGAGTGTGATTC CCAAGACAGACTGCAGGTTACGGCCTGACATCAGAG CCATGGAAAATGGAGAGATAGATCAAGCTAGTGAAG AAAAAAAAACGACTTGAGGAAAAACAAAGAGCAGCCC GCAAAAACAGGTCCAAGTCAGAAGAGGACTGGAAGA CGAGGTGGTTCCATCAAGGTCCTAATCCCTACAATG GAGCACAGGACTGGATTTACTCTGGCAGCTACTGGG ACAGAAATTACTTCAATTTGCCTGACATTTATTAAGGT ACCGCGGGCCCGGGATCCACCGGATCTAGATAA	This paper	ORP1L_Frag2_KpnI
TGGGCGGAGGGTCCGGACTCAGATCTCGAGCTATG GCGTCCATCATGGAAGGGCCGCTGAGCAAATGGAC TAACGTGATGAAGGGCTGGCAGTACCGTTGGTTCGT GCTGGACTACAATGCAGGACTGCTCTCCTACTACAC GTCCAAGGACAAAATGATGAGAGGCTCTCGCAGAGG ATGTGTTAGACTCAGAGGAGCTGTGATTGGTATAGA CGATGAGGACGACAGCACCTTCACAATAACTGTTGA TCAGAAAACCTTCCATTTCCAGGCCCGTGATGCTGA TGAGCGAGAGAAGTGGATCCATGCCTTAGAAGAAAC AATTCTTCGACATACTCTCCAGCTTCAAGGTTTGGAT TCAGGATTTGTTCCTAGTGTCCAAGATTTTGATAAGA AACTTACAGAAGCTGATGCTTACCTACAAATCTTGAT TGAACAATTAAAGCTTTTTGATGACAAGCTTCAAAAC TGCAAAGAAGATGAACAGAGAAAGAAAATTGAAACT CTCAAAGAGACAACAAATAGCATGGTAGAATCAATTA AACACTGCATTGTGTTGCTGCAGATTGCCAAAGACC AGAGTAATGCGGAGAAGCACGCAGATGGAATGATAA GTACTATTAATCCCGTAGATGCAATAT	This paper	XhoI_ORP9L_N_ScaI

CGGAGGGTCCGGACTCAGATCTCGAGCTATGAAGG<br>AGGAAGCATTCCTTAGGCGCAGATTCTCACTTTGTC<br>CTCCGTCCTCTACCCCACAGAAAGTTGACCCACGCA<br>AGCTCACACGAAATCTTCTTCTCTCAGGAGATAATGA<br>GTTGTACCCTCTGTCACCGGGTAAAGACATGGAGCC<br>GAATGGCCCCAGCCTGCCACGAGACGAAGGCCCTC<br>CGACGCCTTCCTCAGCAACAAAGGTGCCACCCGCTG<br>AGTACCGCCTTTGCAATGGGAGTGATAAGGAGTGTG<br>TAAGTCCCACTGCAAGAGTCACAAAAAAAGAAACACT<br>CAAGGCCCAGAAAGAGAACTACCGCCAAGAAAAGAA<br>GCGCGCTACCAGGCAGCTCCTTTCAGCACTCACTGA<br>TCCATCTGTGGTCATAATGGCAGATTCCTTGAAAATA<br>AGGGGTACTCTTAAAAGCTGGACAAAACTGTGGTGT<br>GTCCTGAAGCCTGGGGTTTTGCTGATTTATAAGACG<br>CCCAAAGTAGGTCAATGGGTGGGCACAGTCCTTCTC<br>CATTGTTGTGAACTCATTGAACGGCCATCTAAGAAAG<br>ACGGATTTTGTTTCAAACTCTTTCATCCATTGGATCA<br>GTCCGTTTGGGCCGTTAAGGGTCCTAAGGGAGAATC<br>AGTAGGGAGCATCACGCAGCCGCTTCCTTCCTCTTA<br>TTTGATCTTCAGGGCCGCAAGCGAGAGCGATGGCC<br>GATGCTGGCTTGACGCCCTGGAGCTGGCCCTTAGAT<br>GCAGCTCTCTTTTGCGCCTTGGGACGTGCAAGCCTG<br>GGCGCGATGGCGAACCCGGAACTTCCCCAGACGCA<br>TCCCCCTCATCACTCTGCGGACTTCCAGCGAGCGCC<br>ACTGTTCATCCAGACCAGGACCTTTTTCCCCTCAACG<br>GGAGCAGTCTTGAAAACGATGCATTTAGTGATAAGA<br>GCGAGAGGGAAAATCCAGAAGAATCAGACACAGAAA<br>CTCAAGACCACTCCCGGAAAACAGAATCCGGCAGCG<br>ACCAAAGCGAGACCCCTGGCGCGCCCGTCCGCCGA<br>GGGACAACCTACGTCGAACAAGTGCAAGAAGAGCTG<br>GGTGAACTCGGAGAGGCTTCCCAAGTGGAGACTGT<br>GTCTGAGGAAAACAAGTCACTTATGTGGACATTGCTT<br>AAGCAACTGAGGCCAGGTATGGACCTCAGTAGGGTC<br>GTGCTTCCAACGTTCGTACTTGAGCCGCGATCATTT<br>CTGAACAAACTTAGCGACTACTATTACCACGCGGAT<br>CTCCTGAGCAGGGCAGCTGTAGAAGAAGACGCCTA<br>CAGTAGAATGAAATTGGTGCTGCGCTGGTACCTGAG<br>CGGTTTCTACAAGAAGCCCAAGGGCATTAAGAAGCC<br>TTACAATCCAATATTGGGCGAGACGTTTAGATGTTGT<br>TGGTTTCATCCTCAGACAGACAGCAGAACTTTTTATA<br>TTGCAGAACAGGTCTCTCATCACCCCCCGGTAAGTG<br>CGTTTCATGTTTCCAACAGGAAAGATGGTTTCTGCAT<br>AAGTGGTAGCATAACGGCCAAAAGCCGCTTCTACGG<br>CAATTCTCTCTCTGCTTTGCTTGATGGGAAAGCCACT<br>CTTACGTTTCTCAATCGAGCAGAGGATTATACATTGA<br>CAATGCCCTATGCCCATTGCAAAGGGATTTTGTATG<br>GCACAATGACACTTGAGCTGGGAGGTAAAGTAACCA<br>TCGAGTGCGCAAAAAATAACTTCCAGGCACAGTTGG<br>AGTTCAAACTTAAGCCATTTTTCGGAGGATCCACATC<br>CATCAATCAAATTAGCGGTAAAATAACGTCCGGTGA<br>GGAAGTTCTTGCCTCTCTCTCTGGACACTGGGACCG<br>GGACGTATTCATAAAAGAAGAAGGCAGTGGATCAAG<br>TGCACTCTTCTGGACCCCATCAGGTGAAGTTCGACG<br>ACAAAGATTGCGACAACACACCGTCCCACTGGAAGA<br>GCAAACCGAGCTTGAATCTGAAAGACTTTGGCAACA<br>CGTCACTAGGGCTATCTCTAAAGGGGATCAGCATCG<br>GGCCACGCAGGAAAAGTTCGCCCTCGAAGAAGCTC<br>AAAGGCAAAGGGCTAGAGAACGACAAGAGTCTTTGA<br>TGCCGTGGAAGCCTCAACTGTTCCACCTCGATCCGA<br>TAACCCAAGAATGGCATTACAGGTATGAGGACCACT	This paper	XhoI-ORP5-HindIII

CACCCTGGGACCCCCTCAAAGACATTGCCCAGTTCG<br>AGCAGGATGGGATATTGCGAACACTCCAACAAGAGG<br>CTGTAGCAAGACAAACCACCTTTTTGGGGTCTCCCG<br>GACCAAGACACGAACGAAGCGGGCCCGATCAAAGA<br>CTTCGAAAAGCCTCCGATCAACCATCTGGCCACTCC<br>CAAGCAACCGAGAGCAGCGGAAGTACCCCGGAGAG<br>CTGCCCGGAATTGTCAGACGAGGAACAGGACGGCG<br>ACTTTGTGCCAGGGGGTGAATCTCCTTGCCCGAGGT<br>GTCGCAAAGAGGCGAGACGCCTGCAGGCATTGCAT<br>GAAGCCATCCTTTCTATCAGGGAGGCTCAACAGGAA<br>CTTCATCGCCACCTCTCCGCAATGTTGTCTAGTACG<br>GCGCGGGCCGCACAAGCTCCGACACCCGGACTGTT<br>GCAGTCACCGAGGAGCTGGTTCCTCTTGTGCGTTTT<br>CCTCGCATGTCAACTTTTTATCAACCACATTCTGAAG<br>TAGCAAGCTTCGAATTCTGCAGTCGACGGTACCGCG<br>GGCCCGGGATCC		

TCCGGACTCAGATCTCGAGCTATGAGTTCAGATGAG<br>AAGGGCATTTCCCCTGCTCATAAAACATCCACTCCAA<br>CCCATAGAAGTGCCTCCTCTTCAACATCCTCCCAAA<br>GGGACAGTAGGCAGAGTATTCACATACTGGAGAGGA<br>CTGCTTCCTCTAGCACCGAGCCCTCTGTAAGTCGGC<br>AATTGCTAGAACCGGAGCCAGTCCCCCTCTCCAAGG<br>AAGCTGACAGCTGGGAAATTATAGAAGGGCTGAAAA<br>TAGGCCAAACCAATGTCCAGAAACCAGACAAACATG<br>AGGGCTTTATGCTGAAGAAAAGAAAATGGCCTTTAAA<br>AGGCTGGCACAAGCGTTTTTTTGTCCTGGATAATGG<br>AATGTTAAAGTATTCAAAGGCACCACTCGATATTCAG<br>AAAGGAAAGGTCCATGGGAGCATAGATGTGGGACTC<br>TCAGTCATGTCAATTAAAAAGAAAGCTCGAAGAATAG<br>ACCTTGACACCGAAGAGCACATCTATCATTTGAAGGT<br>GAAATCCCAGGACTGGTTTGATGCATGGGTCTCCAA<br>ACTGCGACATCATCGGTTGTATCGTCAGAATGAAATT<br>GTGAGATCACCAAGAGATGCTAGTTTTCACATATTTC<br>CTTCAACGTCCACAGCTGAATCCTCACCAGCTGCTA<br>ATGTTTCTGTAATGGATGGAAAGATGCAACCAAACAG<br>CTTTCCGTGGCAGTCCCCTTTACCATGCAGCAATAG<br>CCTCCCTGCAACGTGCACAACTGGCCAGAGTAAAGT<br>GGCAGCCTGGTTACAGGACTCGGAAGAGATGGACA<br>GGTGTGCAGAAGATCTTGCACATTGCCAGTCAAACC<br>TTGTGGAACTTAGCAAACTCCTGCAAAATTTGGAAAT<br>ACTTCAGAGAACTCAGTCAGCACCTAACTTTACTGAC<br>ATGCAGGCTAACTGTGTAGATATTTCAAAGAAAGACA<br>AGCGGGTCACAAGACGATGGAGAACAAAAAGTGTCA<br>GCAAAGATACAAAAATACAACTGCAGGTTCCTTTCAG<br>TGCTACCATGTCACCAGTTCGCTTGCATTCCTCCAAC<br>CCCAACCTTTGTGCAGATATTGAATTTCAGACTCCCC<br>CTAGCCACCTCACTGACCCTCTGGAAAGTTCAACAG<br>ATTATACAAAGCTGCAAGAAGAATTTTGTCTAATCGC<br>ACAGAAAGTGCATTCTCTTTTGAAGTCTGCATTTAAT<br>AGCATAGCTATAGAGAAGGAGAAGCTGAAGCAGATG<br>GTTTCCGAGCAGGATCACAGTAAAGGCCACAGCACG<br>CAGATGGCACGGCTCCGACAGTCACTGTCTCAGGCA<br>CTCAACCAGAATGCTGAACTAAGGAGTCGGTTGAAC<br>AGAATACATTCAGAGTCTATTATTTGTGATCAGGTTG<br>TCAGTGTAAATATTATTCCTAGCCCTGATGAGGCTGG<br>TGAGCAAATCCATGTCAGTCTCCCCTTATCACAGCAA<br>GTAGCCAATGAGAGCCGCCTCTCCATGTCAGAGTCT<br>GTTTCTGAGTTCTTTGATGCCCAAGAGGTGCTCCTCT<br>CTGCAAGTTCGTCAGAGAATGAGGCTTCAGATGATG<br>AGTCTTACATCAGTGATGTGAGTGATAATATATCTGA<br>AGACAACACCAGTGTTGCAGACAATATTTCTCGGCA<br>AATCCTGAATGGGGAGCTTACAGGAGGGGCCTTCC<br>GAAATGGGCGTCGAGCATGCCTGCCAGCTCCTTGTC<br>CTGACACCAGTAACATTAACCTGTGGAATATCTTGAG<br>GAACAACATTGGTAAAGACCTGTCTAAAGTCTCTATG<br>CCTGTGGAGCTAAACGAGCCGCTCAACACCCTGCAG<br>CACCTCTGTGAGGAAATGGAATACAGCGAGCTCCTG<br>GACAAGGCTTCGGAAACTGATGATCCATATGAGCGC<br>ATGGTTCTCGTTGCCGCATTTGCAGTTTCAGGATACT<br>GCTCCACCTATTTCAGAGCAGGAAGTAAGCCATTCA<br>ACCCAGTCCTTGGGGAGACTTATGAATGCATTAGAG<br>AAGACAAGGGATTCCGCTTTTTCTCAGAACAGGTTA<br>GCCATCATCCACCCATTTCTGCCTGTCACTGTGAATC<br>AAAGAATTTTGTGTTTTGGCAAGATATCAGATGGAAA<br>AACAAGTTCTGGGGGAAGTCGATGGAAATCCTGCCT<br>GTTGGAACACTGAATGTCATGCTTCCAAAGTATGGA	This paper	XhoI-ORP6-KpnI

GATTACTATGTGTGGAATAAAGTCACCACTTGCATAC ACAACATCCTCAGTGGGAGAAGATGGATAGAACATT ATGGAGAAGTAACCATCAGAAATACCAAAAGCAGTG TTTGCATTTGCAAACTCACATTTGTCAAGGTGAATTA TTGGAATTCTAACATGAATGAAGTCCAGGGGGTGGT GATAGATCAGGAGGGGAAGGCGGTGTACCGGCTGT TTGGAAAGTGGCATGAAGGACTCTACTGTGGTGTGG CCCCCTCTGCAAAGTGCATTTGGAGACCAGGTTCCA TGCCAACAAACTATGAGCTGTACTATGGCTTCACAAG GTTTGCTATTGAGCTCAATGAGTTAGATCCAGTACTA AAAGATCTCCTTCCACCAACAGACGCCCGGTTCCGG CCAGATCAAAGATTTTTGGAAGAAGGAAATTTAGAAG CTGCAGCATCAGAGAAGCAAAGAGTAGAGGAACTCC AGAGATCTCGGAGACGATATATGGAAGAAAACAATC TTGAACATATACCAAAATTTTTTAAAAAAGTTATTGAT GCCAATCAAAGAGAAGCCTGGGTTTCTAACGACACC TACTGGGAGCTTCGAAAGGACCCTGGGTTTAGCAAA GTAGACAGCCCTGTTCTTTGGTAGGGTACCGCGGGC		
**Recombinant DNA**		
SiT-N-mCherry	Addgene	55133
mScarlet-Giantin-C	Addgene	85048
iRFP-FRB-Giantin	Addgene	139313
mCherry-P4M	Addgene	51471
TGN38 EGFP	Addgene	128148
SAC1deltaTMD-FKBP-mCherry	Addgene	108123
pORANGE Cloning template vector	Addgene	131471
Lck-mScarlet-I	Addgene	98821
pLJM1-EGFP	Addgene	19319
pMD2.G	Addgene	12259
pRSV-REV	Addgene	12253
pMDL/pRRE	Addgene	12251
mCherry-C1	De Camilli lab	YS300
mRuby-C1	De Camilli lab	YS286
iRFP-P4M	De Camilli lab	YS313
EGFP-GRAM$_{1b}$ (G187L) (EGFP-GRAM-H)	Ercan et al (2021)	BE84
pLJM1-EGFP-GRAM$_{1b}$ (G187L) (EGFP-GRAM-H)	Ercan et al (2021)	TN209
pNIC28-Bsa4 mCherry-D4H	Ercan et al (2021)	D148
mCherry-GRAM-H	Ercan et al (2021)	TN200
mRuby-GRAMD1b	Naito et al (2019)	LK26
mRuby-OSBP	Naito et al (2019)	D42
mRuby-ORP4	Naito et al (2019)	D35
PM-FRB-tagBFP	Naito et al (2019)	TN250
miRFP-FKBP-GRAMD1b	Naito et al (2019)	TN60
miRFP-FKBP-GRAMD1b (5P)	Naito et al (2019)	TN67
mRuby-GRAMD1b (5P)	Naito et al (2019)	TN44
TPST2-GFP	Spooner et al (2008)	TN388
EGFP-GOLPH3	Tie et al (2022)	TN384
Vamp4-GFP	Tran et al (2007)	TN386
pORANGE_ORP9_4_intermidiate	This paper	TN330
pORANGE_ORP9_4_CT	This paper	TN334
pORANGE_intermediate_OSBP_2	This paper	TN353
pORANGE_mScarlet-I-OSBP_site_2	This paper	TN359
mRuby-ORP1	This paper	D32
mRuby-ORP2	This paper	D33

mRuby-ORP3	This paper	D34
mRuby-ORP5	This paper	TN416
mRuby-ORP6	This paper	TN417
mRuby-ORP7	This paper	D38
mRuby-ORP8	This paper	TN137
mCherry-ORP9	This paper	TN219
mCherry-ORP10	This paper	TN142
mCherry-ORP11	This paper	TN140
EGFP-ORP9	This paper	TN152
EGFP-ORP9 (1-113)	This paper	HN005
EGFP-ORP9 (1-195)	This paper	HN012
EGFP-ORP9 (1-282)	This paper	TN294
EGFP-ORP9 (196-282)	This paper	HN007
EGFP-ORP9 (100-328)	This paper	TN292
EGFP-ORP9 (283-736)	This paper	HN019
EGFP-ORP9 (329-736)	This paper	HN009
EGFP-ORP9 (Δ114-195)	This paper	HN026
EGFP-ORP9 (100-736)	This paper	TN389
EGFP-ORP9 (FY/AA)	This paper	TN258
EGFP-ORP9 (R22E)	This paper	TN391
mCherry-VAPA	This paper	HN020
mCherry-VAPB	This paper	HN021
mCherry-FKBP-ORP9L_329-736	This paper	TN255
Hairpin-mCherry-FKBP-ORP9L_329-737	This paper	TN289
pLJM1-ER-mCherry-FKBP-ORP9	This paper	TN314
pLJM1-ER-mCherry-FKBP-ORP9 (HH/AA)	This paper	TN323
mCherry-ORP9 (HH/AA)	This paper	TN244
mCherry-ORP9 (AAA)	This paper	TN378
SP-tagBFP-TGN38_C	This paper	TN316
tagBFP-TGN38-FRB	This paper	TN322
pLJM1-ER-mCherry-FKBP-OSBP	This paper	TN341
PX459-ORP9_sgRNA_#1	This paper	TN157
PX459-ORP9_sgRNA_#2	This paper	TN158
mCherry-OSBP	This paper	D184
EGFP-D4H	This paper	LK59
mRuby-PH-GRAMD1b	This paper	TN429
**Software and Algorithms**		
Prism	Graph Pad	
PyMoL	The PyMOL Molecular Graphics System, Version 1.2r3pre, Schrödinger, LLC.	
ImageJ Fiji	Schneider et al (2012)	https://imagej.nih.gov/ij/
Imaris	Oxford instruments	
