# Supplementary material for: Regulation of cellular cholesterol distribution via non-vesicular lipid transport at ER-Golgi contact sites
Source: Nat Commun. 2023 Sep 21;14:5867. doi: 10.1038/s41467-023-41213-w (PMC10514280; doi:10.1038/s41467-023-41213-w)
Supplement: Supplementary file 3 — Description of Additional Supplementary Files [file 41467_2023_41213_MOESM3_ESM.pdf]

# Description of Additional Supplementary Files

**File name: Supplementary Movie 1**

**Description: The effect of PI4P depletion by PIK93 on TGN localization of endogenous ORP9 and OSBP**

A live HeLa cell in which mNeonGreen was tagged to the C-terminus of ORP9 (endoORP9-mNG) (left) and mScarlet-I was tagged to the N-terminus of OSBP (mSc-endoOSBP) (middle) expressing iRFP-tagged P4M (iRFP-P4M) (PI4P biosensor) (right) was imaged under SDC microscopy. Images were taken every 30 seconds, and PIK93 (final concentration: 250 nM) was added at 10 min time point. The region around the Golgi is shown. Image size, 11.0 μm x 11.0 μm

**File name: Supplementary Movie 2**

**Description: Rapamycin-induced acute recruitment of ORP9 to the TGN in QKO cells**

A live QKO HeLa cell expressing ER-mCherry-FKBP-ORP9 (left; magenta) and tagBFP-TGN38-FRB together with iRFP-P4M (PI4P biosensor) (middle; grayscale) and EGFP-GRAM-H (accessible cholesterol biosensor) (right; green) was imaged under SDC microscopy. Images were taken every 1 minute, and rapamycin (final concentration: 200 nM) was added at 10 min time point. The region around the Golgi is shown. Image size, 6.6 μm x 6.6 μm

**File name: Supplementary Movie 3**

**Description: Rapamycin-induced acute recruitment of Sac1 to the TGN in QKO cells**

A live QKO HeLa cell expressing mCherry-tagged Sac1ΔTM (PI4P phosphatase domain of Sac1) fused with FKBP module [mCherry-Sac1ΔTM-FKBP] (left; magenta) and tagBFP-TGN38-FRB together with iRFP-P4M (PI4P biosensor) (middle; grayscale) and EGFP-GRAM-H (accessible cholesterol biosensor) (right; green) was imaged under SDC microscopy. Images were taken every 1 minute, and rapamycin (final concentration: 200 nM) was added at 10 min time point. The region around the Golgi is shown. Image size, 6.6 μm x 6.6 μm

**File name: Supplementary Movie 4**

**Description: Rapamycin-induced acute recruitment of GRAMD1b to the TGN in QKO cells**

A live QKO HeLa cell expressing miRFP-FKBP-GRAMD1b (left; magenta) and tagBFP-TGN38-FRB together with iRFP-P4M (PI4P biosensor) (middle; grayscale) and EGFP-GRAM-H (accessible cholesterol biosensor) (right; green) was imaged under SDC microscopy. Images were taken every 1 minute, and rapamycin (final concentration: 200 nM) was added at 10 min time point. The region around the Golgi is shown. Image size, 6.6 μm x 6.6 μm
