# Supplementary material for: Regulation of cellular cholesterol distribution via non-vesicular lipid transport at ER-Golgi contact sites
Source: Nat Commun. 2023 Sep 21;14:5867. doi: 10.1038/s41467-023-41213-w (PMC10514280; doi:10.1038/s41467-023-41213-w)
Supplement: Supplementary file 8 — Reporting Summary [file 41467_2023_41213_MOESM8_ESM.pdf]

nature portfolio

Corresponding author(s): Yasunori Saheki

Last updated by author(s): Aug 16, 2023

Reporting Summary

Nature Portfolio wishes to improve the reproducibility of the work that we publish. This form provides structure for consistency and transparency in reporting. For further information on Nature Portfolio policies, see our Editorial Policies and the Editorial Policy Checklist.

Statistics

For all statistical analyses, confirm that the following items are present in the figure legend, table legend, main text, or Methods section.

n/a	Confirmed	
☐	☒	The exact sample size (*n*) for each experimental group/condition, given as a discrete number and unit of measurement
☐	☒	A statement on whether measurements were taken from distinct samples or whether the same sample was measured repeatedly
☐	☒	The statistical test(s) used AND whether they are one- or two-sided<br>*Only common tests should be described solely by name; describe more complex techniques in the Methods section.*
☒	☐	A description of all covariates tested
☐	☒	A description of any assumptions or corrections, such as tests of normality and adjustment for multiple comparisons
☐	☒	A full description of the statistical parameters including central tendency (e.g. means) or other basic estimates (e.g. regression coefficient) AND variation (e.g. standard deviation) or associated estimates of uncertainty (e.g. confidence intervals)
☐	☒	For null hypothesis testing, the test statistic (e.g. *F*, *t*, *r*) with confidence intervals, effect sizes, degrees of freedom and *P* value noted<br>*Give P values as exact values whenever suitable.*
☒	☐	For Bayesian analysis, information on the choice of priors and Markov chain Monte Carlo settings
☒	☐	For hierarchical and complex designs, identification of the appropriate level for tests and full reporting of outcomes
☒	☐	Estimates of effect sizes (e.g. Cohen's *d*, Pearson's *r*), indicating how they were calculated

Our web collection on statistics for biologists contains articles on many of the points above.

Software and code

Policy information about availability of computer code

Data collection	MetaMorph (SDC microscope) or NIS-Elements (TIRF microscope) was used for the image acquisition. Gen5 3.09 was used for collecting data for cholesterol oxidase assay. Image Lab was used for collecting data for immunoblotting and amphotericin B resistance assay
Data analysis	Fiji (ImageJ) software or Imaris was used for image processing, Prism 9 (Graphpad) was used for statistical analysis, AlphaFold Protein Structure Database was used for structure prediction and PyMOL was used for structural modelling.

For manuscripts utilizing custom algorithms or software that are central to the research but not yet described in published literature, software must be made available to editors and reviewers. We strongly encourage code deposition in a community repository (e.g. GitHub). See the Nature Portfolio guidelines for submitting code & software for further information.

Data

Policy information about availability of data

All manuscripts must include a data availability statement. This statement should provide the following information, where applicable:

- Accession codes, unique identifiers, or web links for publicly available datasets
- A description of any restrictions on data availability
- For clinical datasets or third party data, please ensure that the statement adheres to our policy

The authors declare that the data supporting the findings of this study are available within the paper and its supplementary information file. Source Data file for Figures 1b, 1g, 2i, 3a, 3d, 3f, 3h, 4b-c, 4e, 5b, 5d, 5f, 6b, 6d, 6h, 7b,7d, 7f, 8a, 8c-e, 8f, and Supplementary Figures 1b, 1d, 1f, 2a, 2c-f, 2h-m, 2o, 3b, 3d, 3f, 4b, 4d,

5b, 5d, 5f, 6a-c, 6e, 6g, 6i, 6k, 7b, 7e, 8b, 8d, 8f, 9 is provided with this paper. Other data are available from the corresponding author upon reasonable request. Reagents and strains generated for this study are available directly from the authors upon request. The structure for the ORP9 can be accessed from the AlphaFold Protein Structure Database [https://alphafold.ebi.ac.uk/entry/Q96SU4]. All RNA sequencing data are available at the Gene Expression Omnibus accession number GSE240960 [https://www.ncbi.nlm.nih.gov/geo/query/acc.cgi?acc=GSE240960].

Research involving human participants, their data, or biological material

Policy information about studies with human participants or human data. See also policy information about sex, gender (identity/presentation), and sexual orientation and race, ethnicity and racism.

Reporting on sex and gender	Not applicable.
Reporting on race, ethnicity, or other socially relevant groupings	Not applicable.
Population characteristics	Not applicable.
Recruitment	Not applicable.
Ethics oversight	Not applicable.

Note that full information on the approval of the study protocol must also be provided in the manuscript.

Field-specific reporting

Please select the one below that is the best fit for your research. If you are not sure, read the appropriate sections before making your selection.

☒ Life sciences ☐ Behavioural & social sciences ☐ Ecological, evolutionary & environmental sciences

For a reference copy of the document with all sections, see nature.com/documents/nr-reporting-summary-flat.pdf

Life sciences study design

All studies must disclose on these points even when the disclosure is negative.

Sample size	No statistical method was used to predetermine sample size, and the experiments were not randomized for imaging. For imaging experiments, a reasonable number of cells for each experiment (minimum of 10 to maximum of 325 independent cells) were analyzed. For other biochemical and transcriptome assays, at least 3 independent experiments were analyzed.
Data exclusions	No data exclusion was performed for data analysis.
Replication	All attempts of replication for both imaging and biochemical experiments, at least twice for all experiments, were successful.
Randomization	Randomization is not relevant to this study because the cells / samples in a particular condition did not exhibit differences in appearances. All cells / samples were analyzed in the same way.
Blinding	Blinding is not relevant to this study because the cells / samples in a particular condition did not exhibit differences in appearances. All cells / samples were analyzed in the same way.

Reporting for specific materials, systems and methods

We require information from authors about some types of materials, experimental systems and methods used in many studies. Here, indicate whether each material, system or method listed is relevant to your study. If you are not sure if a list item applies to your research, read the appropriate section before selecting a response.

Materials & experimental systems

n/a	Involved in the study
☐	☒ Antibodies
☐	☒ Eukaryotic cell lines
☒	☐ Palaeontology and archaeology
☒	☐ Animals and other organisms
☒	☐ Clinical data
☒	☐ Dual use research of concern
☒	☐ Plants

Methods

n/a	Involved in the study
☒	☐ ChIP-seq
☒	☐ Flow cytometry
☒	☐ MRI-based neuroimaging

Antibodies

Antibodies used

Anti-ORP9 Sigma-Aldrich/Merck RRID:AB_1854817
Anti-ORP9 Abcam ab151691
Anti-OSBP Sigma-Aldrich/Merck RRID:AB_2676401
Anti-ORP10 Proteintech RRID:AB_2158356
Anti-ORP11 Origene RRID:AB_11124393
Anti-TGN46 BIO RAD RRID:AB_2203291
Anti-TGN46 Proteintech RRID:AB_10597396
Anti-GM130 BD Biosciences RRID:AB_398141
Anti-Actin EMD Millipore RRID:AB_2223041
Anti-SREBP-2 Santa Cruz Biotechnology RRID: AB_2194250
Anti-mNeonGreen Chromotek RRID:AB_2827566
Anti-mCherry Proteintech RRID:AB_2876881
Goat Anti-Rabbit IgG (H+L)-HRP Conjugate Bio Rad RRID:AB_11125142
Goat Anti-Mouse IgG (H+L)-HRP Conjugate Bio Rad RRID:AB_11125547
Donkey anti-Sheep IgG (H+L) Cross-Adsorbed Secondary Antibody, Alexa Fluor™ 594 Thermo Fisher Scientific RRID:AB_10562537

Validation

Anti-ORP9 (Sigma-Aldrich/Merck), Immunoblotting, http://antibodyregistry.org/AB_1854817 and Figure 3a in the manuscript
Anti-ORP9 (Abcam), Immunolabelling, Figure 3b in the manuscript
Anti-OSBP (Sigma-Aldrich/Merck), Immunoblotting and Immunolabelling, http://antibodyregistry.org/AB_2676401
Anti-ORP10 (Proteintech), Immunoblotting, https://antibodyregistry.org/AB_2158356 and Supplementary Figure 2j, l in the manuscript
Anti-ORP11 (Origene), Immunoblotting, http://antibodyregistry.org/AB_11124393 and Supplementary Figure 2j, l in the manuscript
Anti-TGN46 (BIO RAD), Immunolabelling, http://antibodyregistry.org/AB_2203291
Anti-TGN46 (Proteintech), Immunolabelling, http://antibodyregistry.org/AB_10597396
Anti-GM130 (BD Biosciences), Immunolabelling, http://antibodyregistry.org/AB_398141
Anti-Actin (EMD Millipore), Immunoblotting, http://antibodyregistry.org/AB_2223041
Anti-SREBP-2 (Santa Cruz Biotechnology), http://antibodyregistry.org/AB_2194250
Anti-mNeonGreen (Chromotek), Immunoblotting and Immunolabelling, http://antibodyregistry.org/AB_2827566
Anti-mCherry (Proteintech), Immunoblotting and Immunolabelling, http://antibodyregistry.org/AB_2876881

Eukaryotic cell lines

Policy information about cell lines and Sex and Gender in Research

Cell line source(s)

HeLa (ATCC®); 293T (ATCC®)

Authentication

The cell line showed characteristic morphology. No further authentication was performed.

Mycoplasma contamination

All cell lines used in this study were tested negative for mycoplasma contamination.

Commonly misidentified lines
(See ICLAC register)

None.
